# Co-receptor signaling in the pathogenesis of neuroHIV

**DOI:** 10.1186/s12977-021-00569-x

**Published:** 2021-08-24

**Authors:** E. A. Nickoloff-Bybel, L. Festa, O. Meucci, P. J. Gaskill

**Affiliations:** 1 Department of Pharmacology and Physiology, Drexel University College of Medicine, 245 N. 15th Street, Philadelphia, PA 19102 USA; 2 Department of Basic and Translational Sciences, School of Dental Medicine, University of Pennsylvania, 240 S. 40th Street, Philadelphia, PA 19104 USA; 3 Department of Microbiology and Immunology, Drexel University College of Medicine, Philadelphia, PA 19102 USA

**Keywords:** HIV, NeuroHIV, CCR5, CXCR4, Co-receptor, Signaling

## Abstract

The HIV co-receptors, CCR5 and CXCR4, are necessary for HIV entry into target cells, interacting with the HIV envelope protein, gp120, to initiate several signaling cascades thought to be important to the entry process. Co-receptor signaling may also promote the development of neuroHIV by contributing to both persistent neuroinflammation and indirect neurotoxicity. But despite the critical importance of CXCR4 and CCR5 signaling to HIV pathogenesis, there is only one therapeutic (the CCR5 inhibitor Maraviroc) that targets these receptors. Moreover, our understanding of co-receptor signaling in the specific context of neuroHIV is relatively poor. Research into co-receptor signaling has largely stalled in the past decade, possibly owing to the complexity of the signaling cascades and functions mediated by these receptors. Examining the many signaling pathways triggered by co-receptor activation has been challenging due to the lack of specific molecular tools targeting many of the proteins involved in these pathways and the wide array of model systems used across these experiments. Studies examining the impact of co-receptor signaling on HIV neuropathogenesis often show activation of multiple overlapping pathways by similar stimuli, leading to contradictory data on the effects of co-receptor activation. To address this, we will broadly review HIV infection and neuropathogenesis, examine different co-receptor mediated signaling pathways and functions, then discuss the HIV mediated signaling and the differences between activation induced by HIV and cognate ligands. We will assess the specific effects of co-receptor activation on neuropathogenesis, focusing on neuroinflammation. We will also explore how the use of substances of abuse, which are highly prevalent in people living with HIV, can exacerbate the neuropathogenic effects of co-receptor signaling. Finally, we will discuss the current state of therapeutics targeting co-receptors, highlighting challenges the field has faced and areas in which research into co-receptor signaling would yield the most therapeutic benefit in the context of HIV infection. This discussion will provide a comprehensive overview of what is known and what remains to be explored in regard to co-receptor signaling and HIV infection, and will emphasize the potential value of HIV co-receptors as a target for future therapeutic development.

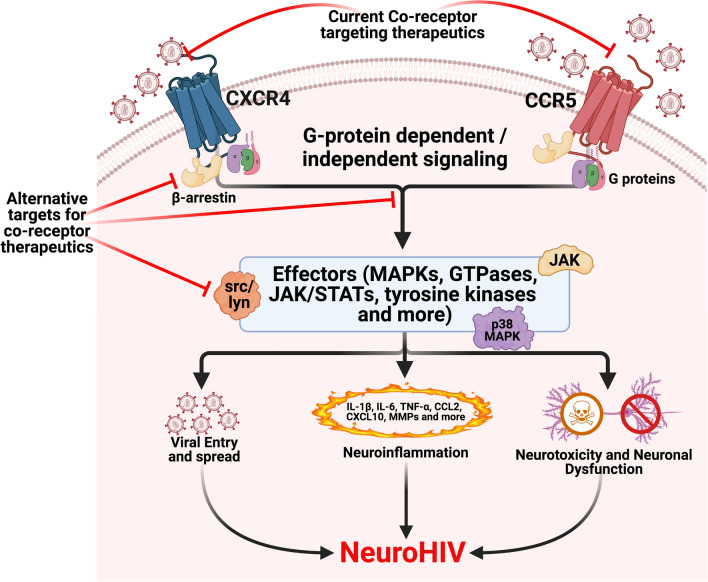

## Introduction

Infection with human immunodeficiency virus (HIV), the cause of acquired immunodeficiency syndrome (AIDS), has been a major public health issue since the emergence of the virus in the early 1980s and more than 38 million people are currently infected with HIV [1]. Today, more than 50% of people living with HIV (PLWH) use combined antiretroviral therapy (cART), and for these individuals, HIV positivity has become a manageable, rather than life threatening, condition. While cART has ameliorated many of the symptoms and comorbidities associated with infection, antiretroviral drugs can only suppress, but not eliminate, viral infection. Suppressed, chronic HIV infection is still associated with a variety of comorbid conditions, including cardiovascular, metabolic and neurological complications [2]. Indeed, 20–50% of infected individuals still suffer from the constellation of neuropathologic, behavioral and cognitive symptoms now known as neuroHIV [3]. Thus, it remains critical to delve further into the neuropathogenesis of HIV to develop novel and more effective therapeutic strategies for the treatment of this disease.

Infection with HIV is almost always mediated by interactions between the HIV envelope protein, gp120, the CD4 receptor and a co-receptor, generally the chemokine receptors CCR5 or CXCR4. These chemokine receptors [4–7], were first associated with HIV infection in the mid-1990’s [8–14]. Initially, the use of CCR5 or CXCR4 was considered cell type specific, with viruses that infected T-cells using CXCR4 and viruses that infected macrophages and microglia using CCR5 [15–17]. This was due, in part, to the use of transformed T-cell lines that predominantly express CXCR4, whereas primary macrophages express more CCR5. Consequently, early researchers classified viruses based on their cell tropism: T-cell (T)-tropic and macrophage (M)-tropic. However, both primary lymphocytes and macrophages express CCR5 and CXCR4 [18–21], and while viruses that infect myeloid cells generally use CCR5 as a co-receptor, this is not an absolute rule [13, 22, 23]. Use of CXCR4 is now generally associated with later stages of infection rather than a specific cell type [24]. Viral tropism is now defined by the co-receptor used for entry; R5 tropic viruses use CCR5, X4 tropic viruses use CXCR4, and viruses that can use either CCR5 or CXCR4 are known as dual-tropic viruses [25, 26]. HIV may be able to use other chemokine receptors as co-receptors [9, 27], but most data indicate CCR5 and CXCR4 are the primary receptors mediating viral entry; therefore, these receptors are the focus of this review.

Much of the current research on CCR5 and CXCR4 focuses on their role as HIV co-receptors. However, both CXCR4 and CCR5 are also chemokine receptors, and their activation mediates complex signaling cascades that initiate a variety of other functions under both pathological and homeostatic conditions [28–30]. As members of the G-protein coupled receptor (GPCR) superfamily, CCR5 and CXCR4 translate ligand binding into intracellular signals through the activation of G proteins. Canonically, both receptors were thought to signal through coupling to G_αi_ [30–33] but there is significant evidence that they also act through G_αq_ and through non-G_α_ protein pathways, including those initiated by G_βγ_ and β-arrestins [29, 34–36]. There has been substantial research into the use of small molecule antagonists, endogenous chemokines and chemokine analogues, and blockade of co-receptor function as HIV therapeutics [37–41]. However, these efforts continue to be hindered by an incomplete understanding of how co-receptor signaling and specific co-receptor conformations contribute to viral infection. Blocking co-receptor activity affects many more processes than just HIV neuropathogenesis, and co-receptor inhibitors often have unwanted side-effects in regard to disruption of homeostatic function [42, 43], further complicating the development of targeted therapeutics for these receptors. There is currently only one FDA-approved antiretroviral targeting an HIV co-receptor, the CCR5 inhibitor Maraviroc, and its efficacy often decreases with disease progression due to mutations that interfere with gp120 binding to CCR5 [37, 44, 45].

Despite these challenges, there is a critical need for novel drugs and strategies that target co-receptor signaling. This is particularly true in the context of neuroHIV, where the interaction of HIV virions with CCR5 and CXCR4 expressed on both myeloid and neuronal cell populations seems to play a role in persistent neuroinflammation and neuronal dysfunction [46–51]. Unfortunately, research into the mechanism(s) by which co-receptor signaling promotes the development of neuroHIV has slowed in recent years, in part owing to the complexity and contradictory data involved in the examination of GPCR signaling. The aim of this review is to reinvigorate this area of research by providing a comprehensive overview of co-receptor signaling and its role in HIV infection, with a specific focus on co-receptor signal transduction and how this influences viral entry, replication, and the pathogenesis of neuroHIV. We will then discuss how substance abuse, which is highly prevalent in the HIV-infected population, alters co-receptor signaling to promote neuropathogenesis. Finally, we will assess the new technologies and recent research in this area, describing the current state of therapeutics specifically targeting co-receptors, and discuss specific experimental questions that are particularly important to the ongoing development of therapeutics capitalizing on co-receptor signaling.

## HIV pathogenesis

While the number of new HIV infections per year has declined since 2000, and people are now living longer with HIV, nearly 2 million new infections and 800,000 HIV-associated deaths still occurred in 2019 [1]. The reductions are due to the evolution of cART, first developed in the mid-1990’s. Prior to cART, HIV infection led to uncontrolled viral replication, loss of CD4 + T-cells and impaired immune function [52, 53]. This left individuals susceptible to opportunistic infections and a rapid progression to AIDS, invariably resulting in death. The use of cART prevents this by suppressing viral replication, leading to a recovery in CD4 + T-cell levels and immune function, although it does not eliminate the virus [52, 54, 55]. Additionally, evidence suggests that viral replication is incompletely suppressed in the CNS [56–60]. Thus, cART is not a cure, although it does dramatically lengthen the quality and quantity of life for chronically HIV infected individuals [3, 54, 55, 61]. Further, the use of cART has also created new health issues, as PLWH suffer from a variety of new comorbidities associated with chronic infection and long-term therapy [62–66].

These comorbidities result from HIV infection and associated inflammation in organs throughout the body. HIV primarily infects CD4 + T-cells and myeloid lineage cells including monocytes and tissue specific macrophages such as microglia and alveolar macrophages [52, 53, 67, 68]. In addition to co-receptors, viral entry requires the CD4 receptor, which is bound by the HIV envelope protein, a dimer of trimers comprised of a trimer of heavily glycosylated gp120 proteins and a connected trimer of gp41 proteins [69, 70]. The interaction between a host CD4 receptor and a gp120 trimer on the external surface of the viral membrane induces a conformational shift in the envelope protein. This exposes further binding sites, allowing gp120 to bind to the viral co-receptors CXCR4 or CCR5 [71, 72], inducing a further conformational shift which causes the trimeric gp41 to “spring out” and insert itself into the host-cell membrane [73, 74]. This initiates membrane fusion, merging the host and viral membranes and enabling the viral capsid, which contains the viral RNA genome and viral proteins such as reverse transcriptase and integrase, to enter the cell [69, 70].

Following entry, the viral capsid undergoes remodeling by host proteins in a process called uncoating, and the viral reverse transcriptase enzyme converts the HIV RNA into proviral DNA, although the precise order of these operations is still not completely clear [75]. There is some debate as to whether the capsid is completely disassembled or remains intact, though more recent evidence supports the latter hypothesis [76–78]. Following uncoating and reverse transcription, the newly generated proviral DNA and additional viral proteins such as reverse transcriptase, Vpr, matrix and integrase proteins, is incorporated into a pre-integration complex made up of a number of cellular proteins as well as the capsid core [76, 79]. This complex then is then transported through a nuclear pore complex into the nucleus, where the viral integrase enzyme then incorporates the proviral DNA into the host genome [79]. In the nucleus, host transcriptional machinery transcribes the viral DNA into mRNA, which is spliced, exported and translated into the early proteins Tat and Rev. These proteins regulate further HIV gene expression, with the Tat protein facilitating transcription elongation and the generation of full-length viral RNA, while the Rev protein facilitates the export of unspliced and incompletely spliced viral mRNA into the cytoplasm to generate the viral polyproteins and accessory proteins [80]. Following translation, individual Gag proteins as well as Gag polyproteins traffic to the plasma membrane where they mediate the essential events in virion assembly within specialized membrane microdomains. During this process, viral envelope proteins are concentrated at the assembly site, the spherical particle is assembled through protein–protein interactions and viral RNA is packaged into the assembling virion [81–83]. In T-cells, this occurs on the inner surface of the plasma membrane, while in macrophages and other myeloid cells it takes place on the surface of internal, plasma membrane connected compartments [84–86]. The viral proteins and viral RNA are incorporated into an immature viral particle that buds off from the membrane [81, 82]. After budding, the viral protease cleaves the viral polyproteins, and the gag proteins assemble into a capsid containing the viral RNA and proteins, forming a mature, infectious virion, which can spread the virus to other cells [81, 82].

### NeuroHIV

Neurological symptoms associated with HIV infection have existed since the start of the epidemic [87, 88], and still affect 20–50% of the infected population despite cART [3, 63, 89, 90]. The presentation and severity of these issues has changed with cART, but infected individuals still suffer from a spectrum of neuropathologic, cognitive, motor, and behavioral sequelae known as HIV-associated neurocognitive disorders (HAND) or neuroHIV [63, 91, 92]. NeuroHIV is initiated by HIV entry into the central nervous system, which occurs rapidly after initial infection [88, 93, 94]. There are several proposed mechanisms by which HIV is thought to enter the brain, but the most widely accepted is the “trojan horse hypothesis” [95], which suggests HIV enters the CNS within infected CD14^+^/CD16^+^ monocytes, and possibly infected CD4 + T-cells, which transmigrate across the blood–brain barrier (BBB) and release virus into the CNS [93, 96–100]. In the brain, HIV targets primarily myeloid lineage cells such as microglia and different populations of CNS macrophages [67, 101, 102]. These infected cells drive the development of neuroHIV through production of new virions as well as the elaboration of inflammatory factors such as cytokines and viral proteins [101–103]. The inflammatory and neurotoxic milieu created by these factors has been proposed to drive neuronal dysfunction and synaptic injury, which in turn is thought to contribute to cognitive deficits observed in patients [47, 48, 104–108]. In addition to myeloid cells, studies also suggest astrocytes may be infected with HIV at low levels [109–111]. It is not clear if astrocyte infection is productive and/or if these cells substantially contribute to viral replication, and there is some controversy as to whether they are infected at all [112–115]. Although central to the development of neurological symptoms, neurons are not infected by HIV [67, 116] and neuronal dysfunction is thought to be initiated by the inflammatory mediators secreted by other infected and activated cells [3].

Prior to cART, HIV-associated neuropathology was characterized by the formation of multinucleated giant cells, presence of microglial nodules, infiltration of lymphocytes, activated CNS macrophages, and neuronal loss [87, 107, 117–120]. These effects were particularly prominent in dopamine-rich brain regions, specifically in the substantia nigra, prefrontal cortex (PFC) and striatal substructures including the caudate nucleus, putamen, and nucleus accumbens [57, 66, 87, 121–125]. Severe behavioral and cognitive symptoms were common [118, 120, 126], and some studies found HIV encephalitis (HIVE) in more than 50% of infected individuals [119, 127]. Viral proteins, including gp120, were observed in the brains of patients, particularly those with HIVE [46, 128, 129], suggesting a neurotoxic role of gp120. A number of studies additionally found CSF viral loads correlated with neurological dysfunction [130–132], further suggesting a link between viral persistence and neuroHIV. With the use of cART, HIVE has almost disappeared, and the symptoms of neuroHIV tend to be subtler, with gradual, non-linear growth over time [3, 126, 133]. NeuroHIV remains prevalent in cART treated individuals despite the suppression of viral replication [90, 104], but the presentation has shifted and overt neuronal loss is no longer readily observed [133]. Instead, PLWH present with hippocampal and cortical changes in synaptic number, reduced frontostriatal connectivity, increases in myeloid cell activation and accumulation of infected myeloid cells, neuroinflammation, oxidative stress, and white matter abnormalities [134–139]. This suggests the etiology of this disease is not solely derived from active viral replication [140–142], although HIV entry into and spread within the CNS is essential to the initiation of neuroHIV.

Current data indicate chronic neuroinflammation is a key factor in neuropathogenesis in cART-treated individuals [138, 143–145], likely resulting from interactions between host CNS cells and the infected cells comprising a stable reservoir of HIV in the brain [146–148]. A number of studies have found markers of immune activation in the plasma [149, 150], CSF [145, 151, 152], and post-mortem brain tissue [142] of cART-treated individuals, even in the absence of detectible virus. Additionally, markers of neuronal injury, including alterations in dendritic spine length and density [106, 153–155], subcortical atrophy [135, 156, 157], and changes in metabolites like N-acetylaspartate [139, 158, 159] are observed, and associated with impaired neurocognition [104–106]. These changes are thought to occur through two distinct, but not mutually exclusive, mechanisms. The direct injury hypothesis suggests that shed viral proteins, including gp120, directly promote neurotoxicity by binding to both CXCR4 and CCR5 on neurons. The bystander effect theory proposes that neuronal injury is caused by the secretion of neuroinflammatory mediators, such as inflammatory cytokines and chemokines, by infected and activated macrophages and microglia [160]. There is substantial debate within the field about these processes, particularly regarding the role of viral proteins in directly promoting neurotoxicity. While in vitro and in vivo rodent studies demonstrate neurotoxic effects of individual viral proteins [49, 106, 128, 161–171], it is not clear whether the concentrations of these proteins produced during cART-treated infection mediate similar effects in the human CNS [172]. Therefore, the source of gp120-mediated coreceptor activity in the CNS in PLWH on cART is not clear and is likely to be the gp120 found on intact virions, rather than shed gp120. Thus, much of the discussion below applies to co-receptors activated through interactions with virions during the process of attachment and entry.

Studies indicate that CNS viral infection is incompletely suppressed by cART, suggesting that neuronal injury and inflammation associated with co-receptor signaling in cART-treated individuals may be due to intact virions. HIV RNA is detectable in the CSF and CNS of cART treated populations, indicating the persistence of viral replication [56–60], although it is not clear if this is directly associated with worsening pathology [56, 60, 173]. This viral persistence may be due to a CNS viral reservoir, likely established well before cART is initiated [174]. These reservoirs are thought to be predominantly composed of myeloid cells, namely perivascular macrophages and microglia, which are long-lived, non-dividing and resistant to HIV-induced apoptosis [96, 175–177]. Due to these properties, as well as the poor CNS penetration of many antiretrovirals [178–181], CNS reservoirs are unaffected by a number of antiretroviral drugs [182], and can produce virus long after initial infection [177]. Given that the interaction of HIV virions with CXCR4 and CCR5 can drive signaling pathways that can directly lead to neuroinflammation and activation of other myeloid populations, it is possible that many of the long-term effects may be due to the activation of co-receptors by viral particles. Taken with the central role for CXCR4 and CCR5 in the spread of the HIV infection throughout the CNS, these co-receptors and the signal transduction processes associated with them make attractive targets for antiretroviral therapeutics targeting both the spread of infection and the development of neuropathogenesis.

## Chemokine receptors

Both CCR5 and CXCR4 are G-protein coupled, 7-transmembrane receptors (GPCR) that belong to a family of 20 proteins known as chemokine receptors. These receptors are expressed on a wide range of cell types and are classified into four subgroups depending on which subfamilies of chemokines they bind: CXC, CC, XC, and CX_3_C receptors [28, 30, 183]. While chemokines are known to act as chemoattractants, chemokine receptor activation also mediates a broad array of physiological processes, including cell migration, cytoskeletal rearrangement, cell survival, and inflammation [28, 184]. These receptors are often upregulated in response to tissue damage or in diseases such as cancer and HIV, attracting immune cells to respond to the damage or insult [30, 185, 186]. While many different chemokines and chemokine receptors play a role in HIV neuropathogenesis [183, 187, 188], CCR5 and CXCR4 are considered the major co-receptors used by HIV-1 strains and are thought to mediate many of the resulting chemokine responses [189, 190], making them appealing targets for antiretroviral development.

Like other GPCRs, chemokine receptors transmit signals resulting from ligand binding via an associated complex of three distinct G-proteins; G_α_, G_β_, and G_γ_ subunits [191]. In the inactive state, GPCRs are coupled to a trimer of these G-proteins, one of each type. Ligand binding initiates a conformational shift in the GPCR, activating the G_α_ subunit by exchanging a bound guanine diphosphate (GDP) for a guanine triphosphate (GTP). This causes the G_α_ subunit to dissociate from the receptor to initiate distinct downstream signaling cascades [192]. There are four distinct subtypes of G_α_ proteins: G_αs_, G_αi_, G_αq_, and G_α12/13_, each associated with distinct signaling cascades, though distinct G-proteins can activate the same downstream effectors or have overlapping functions [191]. The signal transduction pathways initiated by G_α_ proteins are more frequently studied, but G_β_ and G_γ_ subunits, which form an obligate dimer (G_βγ_), can also activate several signaling mechanisms [193–195]. There are also several G-protein independent signaling cascades, which vary with chemokine receptor, cell type and effector [196, 197]. These G-protein independent pathways are mediated by β-arrestins 1 and 2, which can serve as scaffolds for downstream signaling molecules [196, 198–200]. This often involves the recruitment of Src kinases [199, 201], which are also activated by G_αi_ signaling [202], and can lead to downstream activation of mitogen activated protein kinase (MAPK) signaling pathways [203–205] as well as the PI3 kinase (PI3K) signaling pathway [199, 206].

Chemokine receptor signal transduction is also regulated by blocking receptor signaling. This involves GPCR kinases (GRKs) phosphorylating several different residues on the active carboxyl terminus of the GPCR, recruiting β-arrestins [207, 208]. Following their recruitment, β-arrestins carry out two main functions. First, they occlude the G-protein binding site on the chemokine receptor, blocking signaling and desensitizing the receptor [208, 209]. Next, β-arrestins can recruit the cellular machinery required for clathrin-mediated internalization, initiating either receptor recycling [209, 210] or lysosomal degradation of the receptor [211]. Some studies suggest that the strength of the interaction between the arrestin and the receptor regulates this choice, with weaker interactions resulting in degradation and stronger interactions leading to receptor recycling [212]. Irrespective of the precise mechanism, GRK/β-arrestin-mediated desensitization and internalization works in conjunction with ligand stimulation to mediate chemokine receptor signaling.

The primary function of chemokine receptors is to mediate cell migration, and much of the signaling originating from these receptors promotes cell movement. Classically, these receptors couple to G_αi_ [4, 31, 36, 213–215]. Activation of this G protein inhibits adenylate cyclase, decreasing cyclic AMP (cAMP) production [29, 35, 215–217]. G_αi_ can also activate Src and Lyn kinases [202, 218, 219], which in turn activate the small GTPases Ras and/or Raf resulting in MEK and ERK phosphorylation [220–222] and leading to chemotaxis [223–228]. Release of the G_βγ_ subunits can also stimulate cell movement [195, 228, 229], activating PI3K and the serine/threonine kinase AKT [224, 230]; the GTPases Rac and Rho [231]; or phospholipase C-β (PLCβ) [232, 233], which hydrolyzes phosphatidylinositol 4,5-bisphosphate (PIP_2_) to diacylglycerol (DAG) and inositol triphosphate (IP_3_), initiating intracellular calcium (Ca^2+^) release from the endoplasmic reticulum as well as activation of protein kinase C (PKC). Several of these cascades, including activation of PI3K and AKT [199, 206], or Src and ERK1/2 [203–205], can also be triggered by recruitment of β-arrestins and are associated with a number of downstream functions, including chemotaxis and apoptosis [196, 198–200].

These are just some of the vast network of signaling pathways associated with chemokine receptor activation, and both CCR5 and CXCR4 have a number of important pathways specifically associated with their activity. The complexity of these and other signaling networks initiated by co-receptor binding is often amplified by the use of different types of cell systems. The use of different types of cells has enabled more precise interrogation of specific signaling processes, but it has also led to confusing and contradictory data, as co-receptors in distinct cell types often show different responses to similar stimuli. This is particularly problematic when comparing transfected cells and cell lines to primary cells and in vivo systems, as it can create confusion about the physiologically relevant actions of co-receptors during disease. These signaling processes, their downstream effects and the caveats associated with their study are discussed below, and those involved in HIV pathogenesis are described in detail in the subsequent section. Further discussion on the functions and pathways activated by chemokine receptor signaling can be found in additional reviews [29, 34, 234–236].

### CCR5

The CCR5 receptor was first identified as a human monocyte chemokine receptor in 1996, following the discovery of its primary ligands CCL3 (MIP1α), CCL4 (MIP1β), and CCL5 (RANTES) [4–6, 237]. Only CCL4 binds exclusively to CCR5, with CCL3 also binding to CCR1 and CCL5 acting as a ligand for both CCR1 and CCR3 [28, 238]. Several other ligands including CCL2 (MCP-1), CCL7 (MCP-3), CCL8 (MCP-2), CCL13 (MCP-4) and CCL11 (eotaxin), have shown some affinity for CCR5 in vitro in binding studies or competition assays [237], but the in vivo relevance of these interactions is unclear [37, 186]. CCR5 is expressed on a number of cell types, including macrophages [18, 239], microglia [18, 240], T-cells [18, 20, 21], and numerous other immune cells [18] as well as astrocytes [114, 240] and neurons [49, 185, 241].

Under homeostatic conditions, chemokine signaling through CCR5 is associated with numerous physiological processes including leukocyte migration [218, 223, 242–244], regulation of inflammation through cytokine and chemokine release [245, 246], and the activation of cell survival pathways [219]. These functions are thought to be particularly important in the context of viral infection or cellular injury. For example, CCR5 is upregulated during inflammation on CD8 + T-cells, promoting the migration of these cells towards the site of infection and thereby increasing the likelihood of encountering antigen specific cells to enhance the adaptive immune response [247]. This receptor may also play a role in the recruitment of immune cells to the CNS [248], the formation of atherosclerotic plaques [186], and tumor cell migration and survival [249, 250]. These effects require a number of intracellular signaling cascades, some of which may be distinct to CCR5 activation by specific ligands, such as CCL5 [35, 251].

Due to its broad influence, CCR5 signaling is tightly regulated by several processes, generally triggered by phosphorylation of serine residues on its C-terminus and the Asp-Arg-Tyr (DRY) motif leading to GRK/arrestin mediated receptor desensitization and internalization [34, 252]. The early steps of CCR5 desensitization are similar to that of other chemokine receptors and involve C-terminal phosphorylation, recruitment of β-arrestins, and clathrin-dependent endocytosis [34, 207, 253]. Upon internalization, CCR5 is directed through the endosomal recycling compartment to the trans-golgi network (TGN), where it is then recycled back to the cell surface upon resensitization [254, 255]. Importantly, different ligands can induce different fates for CCR5 once it is internalized. Some, like the chemokine analogues PSC-RANTES and AOP-RANTES, promote sequestration of CCR5 in the endosome recycling compartment or TGN [254, 256, 257], while others, like the physiological ligand CCL5, induce recycling back to the cell surface [255, 258]. This appears to be due to the ability of these ligands to alter the structure of the intracellular CCR5 loops via GRK recruitment and/or binding of different β-arrestins [256, 259]. Given that the sequestration of CCR5 is a promising method of blocking viral entry [38, 212, 258, 260–263], further examination of the processes mediating CCR5 desensitization and recycling may have important implications for HIV infection and potential antiretroviral activity.

#### CCR5 signaling

The CCR5 receptor can signal through several distinct G-protein mediated pathways. Signaling through G_αi_ inhibits the activation of adenylate cyclase, regulating the production of cAMP [35, 215, 216, 251] and MAPKs [191, 221, 264], and functions such as T-cell proliferation and chemotaxis [34, 214, 230, 265–267]. Signaling through G_αi_ can be influenced by the formation of oligomers, changing the responses to select ligands [268]. For example, in HEK293 cells co-transfected with CCR5 and/or CCR2, the G_αi_ inhibitor pertussis toxin (PTX) block CCL5-induced Ca^2+^ release in cells expressing only CCR5, but not in cells co-expressing CCR5 and CCR2 and treated with CCL2 and CCL5 [268]. Homodimerization or dimerization with either CCR2 or CXCR4 may also prevent gp120 binding [244, 269, 270], although whether this has any in vivo relevance is unclear.

However, several CCR5 signaling pathways are insensitive to pertussis toxin, indicating the involvement of alternative G-proteins and/or G-protein independent signaling mechanisms [271–273]. Much CCR5-initiated signal transduction can also occur through intracellular Ca^2+^ release and PLCβ activation, which can be mediated by G_βγ_ [32, 215, 219, 232, 233, 242, 274]. Moreover, many studies rely solely on pertussis toxin, which may have G_αi_-independent effects via its B-oligomer [32, 275], or do not make use of specific G-protein inhibitors, making it is difficult to define the specific G-proteins initiating each pathway. Further, while not specific to CCR5, studies have suggested that the release of G_βγ_, rather than G_αi_ is essential for chemotaxis mediated by G_αi_-coupled receptors [195, 229]. Both the PLCβ and PI3K signaling cascades mediate the chemotactic response to CCR5 binding by activating a number of Ser/Thr protein kinases, particularly members of the PAK and FAK families, in macrophages [230, 242, 273, 276] and T-lymphocytes [213, 214, 276]. Signaling via the PLCβ signaling cascade also activates MAPKs [221, 267], which is associated with the production of inflammatory mediators [31, 267, 277] and chemotactic responses [223, 278, 279]. Activation of PI3K is specifically required for CCL5 mediated chemotaxis in macrophages and T-lymphocytes and cytoskeletal rearrangement induced by Rho GTPases [230, 242, 280], and can also activate AKT and MAPK signaling [221, 267]. These data demonstrate the importance of PLCβ and PI3K in CCR5-chemokine signaling, suggesting the release of G_βγ_ may be more critical in mediating the effects of CCR5 than G_αi_.

CCR5 may also couple to G_αq_, which can also initiate IP_3_-mediated intracellular Ca^2+^ release [36, 215, 251, 281]. A recent study showed that both CCL4 and CCL5 could initiate Ca^2+^ flux in HEK.CCR5 cells, and this was inhibited by the G_αq_ inhibitor YM-254,890 [215]. The physiological relevance and extent of G_αq_-specific CCR5 signaling is unclear, due to the extensive overlap between G_βγ_ and G_αq_ signaling. However, the centrality of G_βγ_-mediated PI3K signaling to chemotaxis suggests CCR5 mainly acts through G_αi_ and G_βγ_ in response to physiological ligands [195, 229]. In addition, the binding of HIV gp120 may push CCR5 towards G_αq_ signaling, as siRNA against G_αq_, but not G_αi_, prevented CCR5-mediated changes in viral fusion [281] and gp120 induces several G_αi_ independent signaling effects [273, 282–285].

In addition to G_αi_, G_αq_, and G_βγ_ signaling, several signaling processes may be independent of G-protein activity. Stimulation of CCR5 by either CCL2, CCL3, or CCL5 leads to activation of janus kinases 1 and 2 (JAKs) [214, 271, 272], and activation of JAK2 was insensitive to both pertussis toxin and U73122, an inhibitor of PLCβ [272]. This suggests JAK2 activation is mediated via G-protein independent mechanisms, potentially through a direct JAK-CCR5 interaction, which can lead to the phosphorylation and dimerization of the receptor. Activation of the PI3K/AKT signaling could also be initiated by G-protein independent signaling through β-arrestins, as CCL4 stimulates macrophage chemotaxis by inducing the formation of a β-arrestin signaling complex comprised of PI3K, Pyk2 and Lyn, leading to downstream ERK activation [218].

**Fig. 1:**
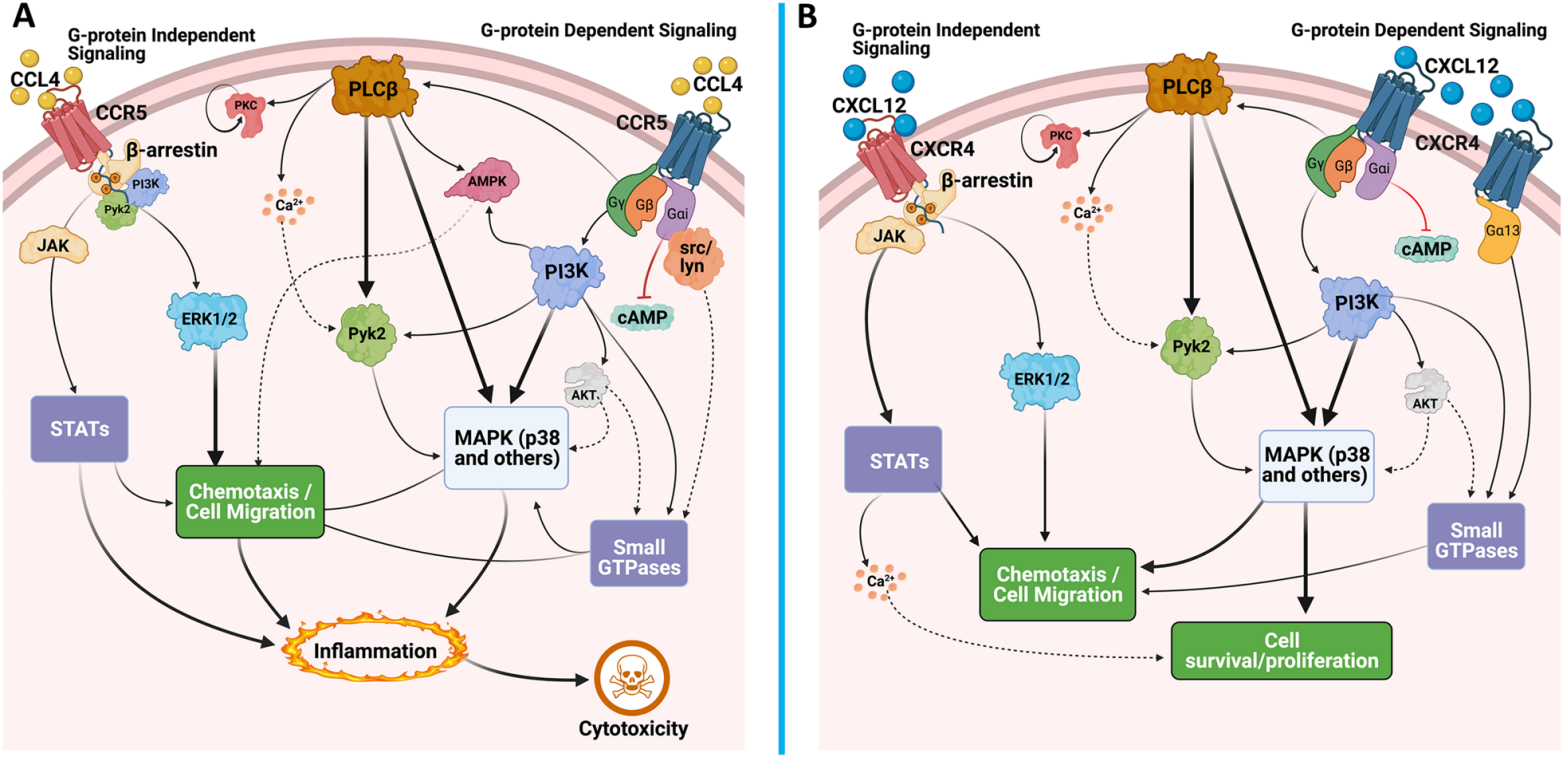
Chemokine signaling through CCR5 and CXCR4. Both CCR5 and CXCR4 can signal through a multitude of pathways, only some of which are represented here. **A** In response to its cognate ligands CCL3, CCL4, and CCL5 (CCL4 shown as a representative ligand), CCR5 can signal through a variety of G-protein dependent and independent pathways. These signaling processes broadly modulate chemotaxis and inflammation. While CCR5 acts through G_αi,_ G_αq_ (not shown) and G_βγ_, the G_βγ_ subunit may regulate the majority of downstream signaling, including PLCβ activation, PI3K activation, and the downstream activation of AMPKs and MAPKs, particularly p38 MAPK. G_αi_ can also interact with Src family kinases, leading to the activation of MAPKs via small GTPase activation, which in turn regulates the chemotactic effects of this receptor. Independent of G proteins, CCR5 signaling can also be mediated through interaction with β-arrestins and the JAK family, activating ERK1/2 and STAT respectively. **B** CXCR4 signaling is also mediated through G_αi_ and G_βγ_, and in certain contexts CXCR4 can also couple to G_α13_. In addition to regulating chemotaxis, CXCR4 signaling also has significant effects on cell survival and proliferation. As with CCR5, PI3K and MAPK activation are central to these signaling pathways and are largely responsible for mediating the effects of CXCL12-CXCR4. The similarities in the signaling pathways between these two receptors highlight how the same effectors can regulate vastly different physiological effects, demonstrating the complexity of studying chemokine receptor signaling. Solid arrows represent defined, published pathways while dashed arrows represent pathways that have not been directly demonstrated but are likely to occur based on the current understanding of GPCR signaling

The responses to CCR5 binding are mediated by a number of effectors, including but not limited to MAPKs [219, 223, 271, 273, 276, 277, 282, 286], signal transducer and activator of transcription (STAT) proteins [214, 272, 287], AMP-activated protein kinases (AMPKs) [242], and small GTPases (Rac, Rho) [266] or FAKs like Pyk2 [213, 243, 272, 273, 276, 282], which play a critical role in chemokine-mediated cellular migration in both lymphocytes and macrophages [223, 230, 243, 266, 276]. Many of these effectors are activated by one or more endogenous CCR5 ligands, such as the phosphorylation of the MAPK ERK 1/2 by both CCL3 and CCL4 [223]. Multiple overlapping pathways are linked to MAPK signaling, including the release of pro-inflammatory chemokines, cell survival, cell death, the activation of STATs, and the activation of matrix metalloproteinases (MMPs) [279, 288, 289]. For example, induction of CCL5 in response to influenza infection leads to G_αi_-mediated activation of both the MEK/ERK and PI3K/AKT signaling cascades, reducing apoptosis in mouse macrophages [219]. Stimulation with CCL5 also leads to Ca^2+^ mobilization and the activation of JAK1/STAT5, triggering cell polarization and migration. However, this did not occur with AOP-RANTES, a synthetic CCL5 derivative that can also bind to CCR5 but does not induce chemotaxis [261, 290], due to differences in the length of G_αi_-association and subsequent release of G_βγ_ [214]. Many of these pathways may also be regulated by PI3K and PLCβ activity. For example, both of these proteins were necessary to promote CCL5-mediated chemotaxis through AMPK in RAW264.7 rodent macrophages [242]. This demonstrates that activation of CCR5 by a single ligand can simultaneously activate several different G-proteins and downstream pathways, regulating multiple cellular functions, a commonality among GPCRs. Indeed, CCR5-mediated activation of JAK/STAT pathways may play a role in T-cell activation and proliferation, although it is unclear whether this is solely due to CCR5 or a combination of CCR receptors, including CCR2 [34, 268, 271, 287, 291]. These data show that CCR5 can initiate signaling through both G-protein dependent and independent pathways and demonstrate the substantial overlap between CCR5 signaling cascades activated by distinct G-proteins (Fig. 1A). These overlaps, combined with the likelihood that the coupling of CCR5 to certain G-proteins and pathways may be different in distinct cell types and species, shows the challenges involved in defining how the specific ligands activate certain signaling pathways and highlights an important area for future studies.

#### Conformation mediated changes in CCR5 signaling

In addition to complications due to overlapping pathways and distinct effects in different cell types [214, 271, 272], analysis of CCR5 signaling is complicated by the existence of conformationally heterogenous populations of CCR5 on the cell surface [257, 292–294]. The conformational differences center on the degree of exposure of binding sites in the N-terminal (NT) and second extracellular loop (ECL2) regions [40, 292], and the interaction of the N-terminus with the transmembrane helix bundles [34, 295]. These differences seem to have a broad impact on receptor function, changing the affinity for different ligands, the coupling to specific G-proteins and the functional potency of 2^nd^ messenger induction [215, 257, 294, 296, 297]. In CCR5 transfected L1.2 lymphocytes, antibodies against the ECL2 region of CCR5 efficiently blocked the binding and functional response of CCL3, CCL4, and CCL5, whereas an antibody against the N-terminal region blocked only CCL4 binding and signaling, not CCL5 [292, 298]. Moreover, an CCR5 antibody mapped to multiple ECL domains was able to increase G_αi_-associated GTPγS binding, but not Ca^2+^ flux, suggesting stabilization of specific conformations of CCR5 is associated with distinct signaling cascades [292].

Other studies show differences in the β-arrestin mediated internalization of specific CCR5 populations, suggesting subpopulations of CCR5 conformations may be resistant to chemokine-induced internalization in macrophages, but not T-cells [299]. This is consistent with studies showing subpopulations of CCR5 may differ between cell types [257, 300]. These differences may involve changes in receptor occupancy based on the ability of different ligands to bind to distinct CCR5 conformations [215]. Notably, natural chemokines only bind to a few distinct conformations, but gp120 may interact with a much wider number of CCR5 populations, potentially due to the interaction of the flexible V3 region of gp120 with the N-terminus of CCR5 [215, 257, 293, 300, 301]. Indeed, changes in the exposure of the NT or ECL2 regions can increase the binding affinity for specific sequences in CCR5 tropic viral envelopes [300], likely playing a role in the maintenance of gp120—CCR5 binding in the presence of natural ligands and some antagonists. Thus, defining not only the CCR5 signaling pathways, but the shifts in ligand binding and signaling associated with those structural changes, could initiate or enhance the development of a number of novel antiretroviral effectors [40, 293, 301, 302].

### CXCR4

The CXCR4 receptor was first identified on peripheral blood lymphocytes in 1994 [7] and is highly expressed in a variety of cell types including lymphocytes, hematopoietic stem cells, neural cells, and stromal fibroblasts [30]. Expression of CXCR4 is relatively low on myeloid cells such as macrophages and microglia [303]. The primary ligand for CXCR4 is the chemokine CXCL12 (SDF-1), which is ubiquitously expressed in both embryonic and adult tissues, including liver, pancreas, spleen, brain, and heart [304]. The CXCL12/CXCR4 signaling axis was long considered monogamous until 2007 when it was discovered that macrophage migration inhibitory factor (MIF) was an alternative ligand for CXCR4 [305]. Additionally, both pharmacologic antagonism and knockdown studies have shown that extracellular ubiquitin (eUb), is also a natural ligand for CXCR4 [306].

In adults, CXCL12-CXCR4 binding promotes the survival and trafficking of immune cells [307, 308]. During embryogenesis, CXCL12 is important for proliferation, migration, and differentiation of immature progenitors [309, 310]. The CXCL12/CXCR4 signaling axis is also important in CNS homeostasis, where it regulates the migration of neural precursors [311, 312], establishment of neural circuitry [313, 314], modulation of NMDA subunit composition [315], and alterations in dendritic spine number and morphology [316, 317]. MIF also plays an important role in cell recruitment and arrest through binding to CXCR2 and CXCR4 [305], and can exert protective functions in liver fibrosis, myocardial ischemia–reperfusion injury and in the developing cerebral cortex upon tissue damage [318]. In contrast to CXCL12, MIF is not associated with homeostatic function, but rather pro-inflammatory and pro-atherogenic activity [305, 319], and is considered an inflammatory cytokine [320]. Similar to other CXCR4 ligands, eUb can also mediate chemotaxis, but the chemotactic activity is weaker than induced by CXCL12 [321]. Furthermore, the interaction of eUb with CXCR4 is independent of the N-terminal receptor domain used by CXC12 and instead relies on binding sites in the 2^nd^ and 3^rd^ extracellular loop [321].

Like CCR5 and other GPCRs, CXCR4 signaling is regulated by desensitization (homologous and heterologous), internalization, and degradation. Direct activation of PKC by phorbol esters [322, 323], T or B cell receptor engagement [324, 325], CXCR1 activation [326], or CCR5 activation [327] are able to induce CXCR4 internalization. CXCR4 can recycle back to the plasma membrane following PKC-mediated internalization [322]; however, the receptor recycles poorly following CXCL12 stimulation [328]. CXCR4 has been shown to be ubiquitinated, sorted to the lysosome, and degraded [329], which is mediated by the E3 ubiquitin ligase AIP4 [330]. Targeting CXCR4 with specific agonists or molecules that promote the internalization and sequestration has not been as widely explored as for CCR5, owing in part to the physiological requirements for CXCR4 binding. However, there has been some progress in finding drugs that can induce signaling while preventing viral binding [43, 331].

#### CXCR4 signaling

Like CCR5 and other chemokine receptors, CXCR4 signals primarily through G-protein dependent pathways, primarily G_αi_ mediated inhibition in cAMP production. Recombinant overexpression systems, such as HEK293T and Sf9 cells show that CXCR4 can activate different G_αi_ proteins, including G_αi1_, G_αi2_, G_αi3_, and G_αo_ in response to CXCL12 stimulation [332], although it seems that CXCR4 couples more efficiently to the G_αi1_ and G_αi2_ rather than G_αi3_ and G_αo_ [333, 334]. Activation of CXCR4 coupled to G_αi_ triggers activation of MAPK and PI3K pathways [335], mediating effects on migration [336–338] as well as cell survival and proliferation [313, 339]. CXCR4 can also act through other G proteins, such as the noncognate G protein G_α13_ [340] or G_αq_ [341, 342], although this may be context specific. CXCR4 only couples to G_αq_ in dendritic cells and granulocytes but not T and B cells [343], and coupling to G_α13_ may have particular relevance in cancer, where G_α13_ is overexpressed [344, 345]. As with CCR5, activation of CXCR4 coupled to G_αq_ can induce IP_3_ mediated Ca^2+^ release through PLCβ [341, 342]; this pathway can also be activated via the released G_βγ_ subunit [235, 346]. Activation of CXCR4 coupled to G_α13_ in Jurkat T cells mediates cell migration via activation of Rho [340], and also mediates CXCR4 trafficking into Rab11 + vesicles during CXCL12-induced endocytosis in T cells [347].

Like CCR5, CXCR4 can also signal through G protein independent mechanisms, such as β-arrestin-mediated signaling. Both β-arrestin-1 and -2 enhance CXCR4-mediated ERK activation [338], and β-arrestin-2 is involved in p38 activation and migration following CXCL12 stimulation [348]. Additionally, upon CXCL12 stimulation, CXCR4 can dimerize and become phosphorylated at intracellular tyrosines by rapid recruitment and activation of JAK2 and JAK3 [349]. This leads to STAT dimerization and activation of the STAT pathway, which is unaffected by pertussis toxin treatment [349]. The JAK/STAT pathway leads to diverse cellular effects, including mobilization of Ca^2+^ from intracellular stores, and after its nuclear translocation, the transcription of several target genes [349]. Studies using a JAK-specific inhibitor have shown that in a T-lymphoblast cell line, the association of G_αi_ with CXCR4 is dependent on JAK, further supporting a co-dependent mechanism between members of the JAK/STAT pathway and G-protein coupled signaling [350] (Fig. 1B). Activation of the JAK/STAT pathway may also be affected by the oligomerization state of this receptor, as homodimerization of CXCR4 is necessary to elicit G protein independent activation of JAK/STAT and enhance the response of CXCR4 to CXCL12 [349]. The heterodimerization/oligomerization of CXCR4 to other receptors may have a number of other potential effects on chemokine signaling responses as well, interfering with the binding to one receptor in the oligomer or altering its ability to interact with or signal through intracellular mediators [351]. This is exemplified by the potential ability of ACKR3 to interact with CXCR4 and affect CXCR4 trafficking and/or coupling to other proteins [352].

## HIV activation of co-receptors

In the context of HIV infection, CCR5 and CXCR4 are primarily studied as co-receptors that interact with gp120 to mediate efficient membrane fusion [16, 70]. In addition to mediating viral entry, gp120 binding also activates CXCR4 and CCR5, but the signaling processes associated with this interaction remains poorly understood. Additionally, it remains unclear if co-receptor signaling is a requirement for efficient viral entry and replication. Early studies in primary CD4 + T-cells, macrophages, and transformed T-cell lines suggested that uncoupling CCR5 from G_αi_ signaling does not alter HIV entry [33, 353–357], but more recent data contradict these findings, showing a requirement for G-protein signaling for viral entry and fusion [281, 358–360], or even post-entry stages of infection [361–366]. At least one recent study also indicates that co-receptor binding by shed or recombinant gp120 provokes different responses than does binding by virus associated gp120 [364], suggesting that some of the difference between studies could be due to the type of gp120 used.

**Fig. 2:**
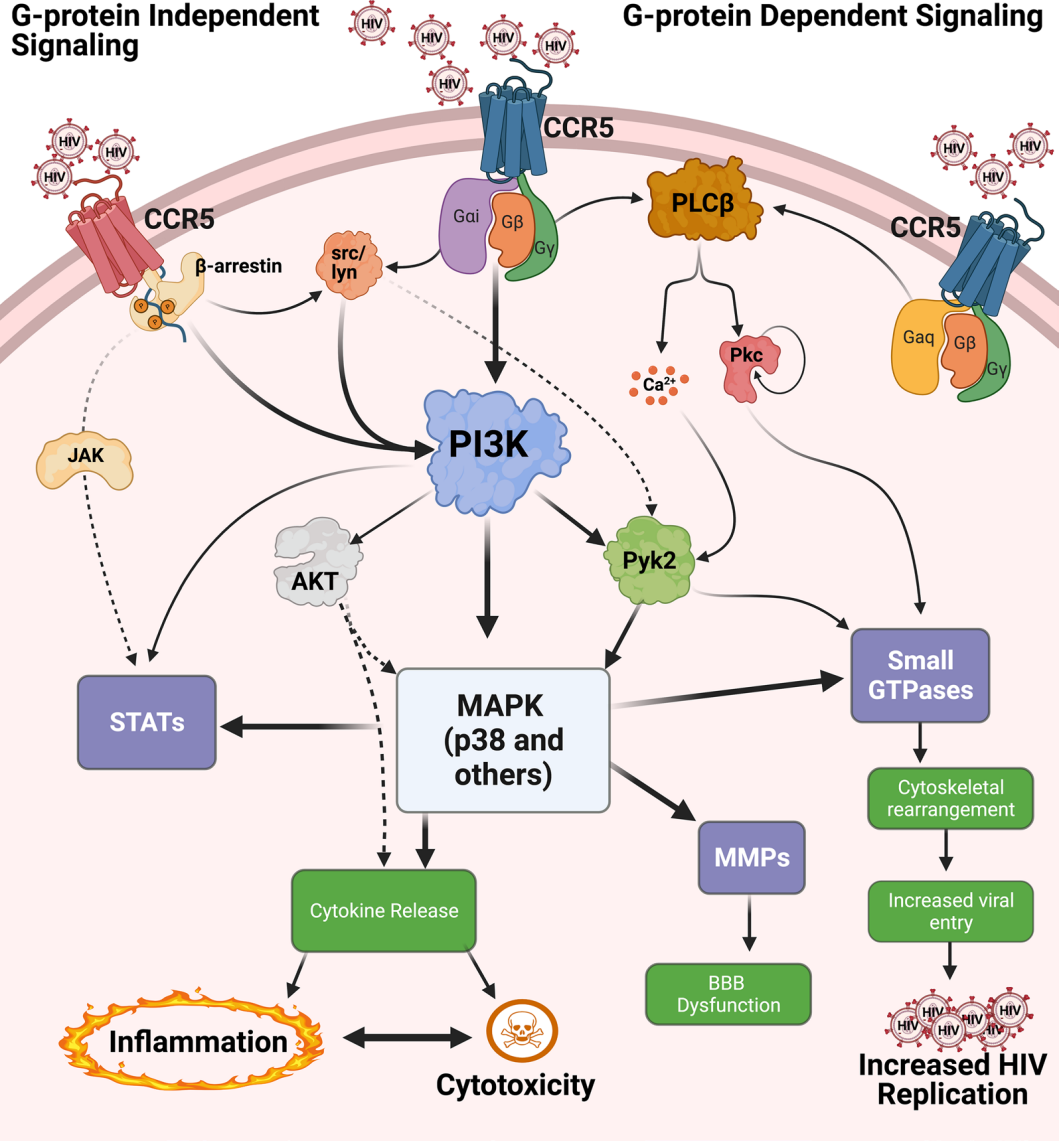
Co-receptor signaling mediated by HIV envelope. The viral envelope glycoprotein gp120 can interact with both CCR5 and CXCR4 during the attachment and entry process, initiating both G-protein dependent and independent signaling. Pathways activated through CCR5-gp120 interactions are shown here as representatives. Activation of both G_αi_ and G_αq_ has been reported in response to gp120. Signaling through G_αq_ mediates activation of PLCβ, calcium release, and downstream activation of small GTPases, which are critical for viral entry, replication, and changes in actin dynamics. Signaling through G_βγ_ can also activate PLCβ, as well as PI3K, both of which are linked to the activation of MAPKs. The most prominent MAPK shown to be involved in these processes is p38 MAPK, although other MAPK are also involved. The activation of MAPKs regulates a large number of downstream pathways, including but not limited to, the activation of STATS, activation of GTPases, the activation of MMPs, and the release of chemokines and cytokines. G_αi_ and G-protein independent interactions with β-arrestins can also activate the Src family of kinases, which are also linked to PI3K and MAPK activation. Activation of all of these pathways can mediate a number of deleterious processes during HIV infection of the CNS, including neuroinflammation, blood–brain barrier dysfunction and increased migration of infected cells to the CNS, increased viral replication, and neurotoxicity. Many of these pathways overlap, meaning that gp120-coreceptor interactions can influence these pathogenic processes through several different, interacting pathways depending on the cell type and system in which they are being studied. Solid arrows represent defined, published pathways while dashed lines indicate pathways that have not been directly demonstrated, but are presumed to occur based on what is known in the literature

While many gp120 induced signaling processes overlap with those initiated by cognate ligands, there are a number of pathways in which the response, kinetics, G-proteins and downstream effectors involved differ from those activated by endogenous ligands. Indeed, relative to endogenous ligands, gp120-co-receptor interactions stimulate the expression of a substantial number of different genes [364, 367]. This section will focus on the most well documented signaling pathways, which are detailed in Fig. 2. In general, we will discuss the effects of gp120, reflecting both the fact that this is the major protein interacting with co-receptors on intact virions and that the literature in the field has historically relied on the use of monomeric gp120. While this provides more precise insight into specific co-receptor driven signaling processes, it is important to note that the concentrations of gp120 used in many of the following studies are unlikely to represent levels in cART-treated individuals. Further, the gp120 driving these processes in the CNS of cART-treated PLWH are likely to present on the virion surface in complex with gp41 and have a different structure than free gp120 [70, 364], and may require interaction with CD4 [71, 74], even if the downstream signaling effects are driven solely by the co-receptors. However, while some studies suggest there may be some differences between the responses of shed gp120 and virus associated gp120 [364], many indicate that monomeric gp120 and virus-associated gp120 largely initiate the same signaling cascades [213, 264, 285, 365, 366, 368–370]. Therefore, we have focused on studies that use gp120 to show clearly defined roles for co-receptors, rather than CD4, in specific signaling mechanisms, to better define specific co-receptor driven signaling processes. Finally, given the extensive overlap between R5 and X4 signaling pathways, they will be discussed together, with specific differences pointed out when necessary.

### Calcium and ion channels

Ca^2+^ flux is important to viral infection [281, 371, 372] and a number of studies have specifically examined the role of Ca^2+^ in HIV entry [281, 373]. In human monocyte derived macrophages (hMDM), both R5 and X4 gp120 can increase intracellular Ca^2+^, although the magnitude of the Ca^2+^ release elicited by X4 gp120 is less than that induced by R5 gp120 [369, 374]. In microglia, this effect is not seen in response to gp41 [375], suggesting it may be exclusive to the gp120 portion of the envelope protein. The response in T-cells is less clear. Some data show that human CD4 + T-cells release Ca^2+^ in response to X4 but not R5 gp120, and that this is dependent on CD4 binding [369]. Other studies show that R5 gp160 from both HIV and SIV increases Ca^2+^ release, but this does not occur in response to X4-tropic envelope proteins [265, 361], although the differences may be due to the specific envelope proteins and cell types used. In cultured rat and human neurons, gp120 appears to directly disrupt neuronal Ca^2+^, via alteration of the [Ca^2+^]_I_ mediated by NMDARs [49, 161], Ca^2+^-gated and Na^+^-gated channels [376]. However, it is unclear whether interactions between gp120 and neuronal CXCR4 and CCR5 contribute to these rises in Ca^2+^, or if the signaling is mediated by interactions between HIV and co-receptors on surrounding glial cells and/or co-receptor independent effects of gp120 [47, 48].

As Ca^2+^ is a prominent 2^nd^ messenger, it is not clear which specific Ca^2+^ activated pathways are associated with HIV entry. However, Harmon et. al. used human U87 astrocytes to show that blocking G_αq_-mediated Ca^2+^ flux by inhibiting ryanodine or IP_3_ receptors blocks cell–cell fusion [281]. Data from our lab show that the dopamine-mediated increase in HIV entry in hMDM requires dopamine-mediated Ca^2+^ release [373], supporting the importance of Ca^2+^ in the entry process. These data suggest that while the two types of gp120 have similar effects on Ca^2+^, the magnitude and therefore outcome of envelope mediated Ca^2+^ release is distinct between X4- and R5- tropic gp120.

G-protein coupled receptors are also associated with ion channel activity [374]. R5-gp120 can suppress the activity of the voltage gated K^+^ channel BEC1 in 293 T cells [377] and X4-tropic envelope can increase the phosphorylation of Kv1.3 channels and induce membrane depolarization in T-cell lines [378]. In human macrophages, X4 and R5 gp120 can elicit Ca^2+^-activated K^+^ currents, Cl^−^ currents, and Ca^2+^-permeant nonselective cation currents, which are blocked by the specific CXCR4 antagonist, AMD3100, or in cells from donors homozygous for the CCR5-$\Delta$ 32 mutation, respectively [374, 379]. This indicates that currents through this channel were mediated specifically by the co-receptors and not CD4 [374]. Ionic signaling via gp120 has also been reported in other cell types as well; exposure of rat primary astrocytes or primary microglia to X4 gp120 stimulated Na^+^/H^+^ antiport and K^+^ conductance [380–382]. Interestingly, there are differences in the ability of natural ligands and gp120 to activate some of these ion channels; whereas non-selective cation channels are activated by R5 and X4 gp120, they were not activated by CXCL12 or CCL4 in hMDM [374]. Further, while the overall pattern of Na^+^ and K^+^ signaling between R5 and X4 gp120 in these hMDM was similar, there were differences in magnitude and kinetics. For example, X4 gp120 induced less frequent K^+^ current activation compared to R5 envelope [374, 379]. Although the precise physiological relevance of these differences is not clear, these studies suggest variability and potentially biased co-receptor signaling in response to different types of gp120, and to gp120 relative to natural ligands.

### Tyrosine and MAP kinase signaling

Downstream of Ca^2+^, gp120 acts on a number of kinases, such as the focal adhesion-related tyrosine kinase Pyk2 [213, 273, 282, 284, 285, 370, 383, 384]. This kinase plays a critical role in R5 gp120 mediated cytokine and chemokine secretion [264, 273, 282–284], dendritic cell migration [285], activation of small GTPases such as Rac1 [281], and activation of MAPKs such as p38 MAPK [273, 282–285, 385]. Pyk2 activation in response to both R5 and X4 gp120 has now been demonstrated in a large number of systems, including primary CD4 + T-cells and T-cell lines [213, 370, 383, 384], hMDM [264, 273, 282–284], dendritic cells [285], and astrocytic models of HIV [281], indicating its importance in gp120 mediated signaling.

In addition to Pyk2, and often downstream of it, gp120 can activate several distinct but often overlapping signaling cascades, several of which are also activated by cognate chemokines of both CCR5 and CXCR4 [273, 277, 286]. These include several different MAPK pathways, such as p38 MAPK [273, 277, 282–285, 386–388], JNK/SAPK [273, 282–284], and ERK cascades [286, 363, 368, 389, 390], all of which can be activated by both R5 and X4 gp120, although the activation of these varies by stimulus and cell type [273, 363, 391]. In hMDM, for example, both types of gp120 can activate p38 MAPK, but X4 gp120 inconsistently induced activation of JNK/SAPK compared to the more consistent activity induced by an R5 envelope [273]. In unstimulated human primary CD4 + T cells, high levels of gp120 increased ERK1/2 phosphorylation, although other studies show ERK1/2 is only activated when T-cells are pre stimulated through the T-cell receptor (TCR) [384, 389, 391]. There is also conflicting evidence regarding ERK1/2 activation by R5 gp120, as studies in both artificial cell systems [277] and primary macrophages [273] reported no effect of R5 gp120 on ERK1/2 phosphorylation. However, others show a robust effect after treatment with gp120 from R5 HIV [273, 286, 363, 390, 392, 393]. Neither differences in cell type nor gp120 strain fully explain these discrepancies in ERK1/2 activity, as several studies used identical cell types and concentrations of gp120 [273, 390]. Given many studies were done with primary human cells, these discrepancies may suggest that population specific heterogeneity plays a significant role in the human signaling response to gp120. It is also unclear whether these signaling events are physiological at the concentrations of gp120 present in vivo as gp120 is often used at extra physiologic levels [172].

Given the importance of Pyk2 and MAPK signaling activation, it is unsurprising that several G-protein-mediated pathways have been implicated in the modulation of Pyk2 and MAPK activity. G_αi_-activated signaling has been linked to PI3K and downstream activation of Pyk2, p38 MAPK, and ERK1/2 [213, 220, 273, 363, 390, 393, 394], while G_αq_ signaling can activate PLCβ and lead to Ca^2+^-mediated activation of Pyk2 [273, 281, 395], as discussed above. Given the variability of gp120 subtypes and cell systems used to define HIV signaling, it is possible that the coupling of CCR5 and/or CXCR4 to specific G-proteins varies with cell type and with X4 or R5 gp120 [36, 197, 215]. Irrespective of the G-proteins involved, data indicate that the activation of the MAPK signaling pathway in response to both R5 and X4 gp120 is a common mediator of gp120 signaling, occurs in a wide variety of cell types, and is linked to a number of downstream pathways that may play a role in the neuropathogenesis of HIV.

Indeed, in many studies, the functional outcome of both R5 and X4 gp120 mediated activation of the MAPK cascade, and in particular, p38 MAPK, has been associated with neuronal dysfunction and death [50, 386, 396–401]. Notably, activation of MAPK signaling cascades by X4 gp120 has been suggested to play a role in the neurotoxic effects of gp120 compared to the protective effects of CXCR4 [160, 167, 400], supporting the concept of differential signaling induced by HIV and chemokine engagement of co-receptors. The clinical relevance of data showing gp120 mediated neurotoxicity is not well understood, and it is unclear if and how R5 and X4 gp120 directly interact with neuronal co-receptors to drive these effects, as neurons lack the CD4 receptor [402]. Further, the amount of neuronal death observed in the brains of cART-treated PLWH has declined substantially [3, 133], suggesting that the current effects of co-receptor activation by virion associated gp120 are not inducing neurotoxicity. Still, the robust amount of data in this area suggests that co-receptor mediated activation of MAPK, specifically p38 MAPK, has an important role in HIV-induced neuropathogenesis.

### HIV-mediated signaling downstream of MAP kinases

Signaling through MAPK regulates a variety of functions, including but not limited to; the release of inflammatory chemokines and cytokines, the activation of STATs, and the activation of MMPs [36, 197, 215, 273, 282, 286, 390, 393, 403, 404]. Secretion of inflammatory cytokines is of particular interest given the central role of these factors in driving HIV-associated neuroinflammation. Exposure to both R5 and X4 envelopes can lead to the production of a large number of chemokines and cytokines, including TNFα, MIP-1α, MIP-1β, IFN-γ, IL-1, IL-6, IL-10, MCP-1, CCL2, and CCL5 [246, 264, 273, 283, 284, 286, 389, 397, 405]. Exposing macrophages and T cells to gp120 also leads to the translocation of several transcription factors critical to cytokine and chemokine secretion, including AP-1, NF-κB, and C/EBP [36, 197, 215, 246, 389, 406]. In addition to promoting inflammation, these chemotactic and inflammatory mediators can modulate HIV replication [407] and recruit uninfected macrophages and T-cells that provide new targets for viral spread.

In hMDM, gp120 mediated release of the inflammatory cytokines IL-1β and TNFα is PI3K dependent [264, 390], although IL-1β secretion was also G_αi_ dependent, suggesting G_αi_-mediated PI3K activation [264]. Both p38 MAPK and ERK1/2 may also mediate the release of these cytokines, potentially through independent signaling pathways [288, 390, 398, 404, 408, 409]. One of these pathways may involve the activation of the Src family of kinases, as Src activation has been linked to R5 gp120 mediated increases in both IL-1β and TNFα in hMDM [264, 286]. Src kinases mediate GPCR-induced phosphorylation of the epidermal growth factor receptor (EGFR) [410], which then activates the PI3K/AKT signaling cascade and/or Pyk2 to mediate MAPK activation [410–412]. Src kinases are also linked to MAPK signaling through β-arrestins and Pyk2, suggesting overlap between G-protein dependent and independent pathways [200, 413]. This suggests that G-protein dependent and independent mechanisms work in concert to promote cytokine and chemokine dysregulation, potentially via the MAPK pathway.

In addition to cytokines, STATs are also upregulated in response to R5 and X4 gp120 [403, 414–416]. Canonically, activation of these latent cytoplasmic transcription factors is mediated by JAKs [417–419] in response to inflammatory and regulatory stimuli [419, 420], although JAK independent mechanisms have been described [403, 415, 421]. Gp120-mediated activation of STATs 1 and 3 leads to activation of IL-6 in monocyte derived dendritic cells (MDDC) and HBMECs [403, 414, 415], although the mechanisms leading to this activation differed. In MDDCs, activation involved p38 MAPK and activation of NF-κB [403], whereas in HBMECs it involved activation of PI3K [414, 415]. In MDDC, the actions of gp120 were distinct from those of CCL4, which did not induce STAT3 activation [403], although other studies have found that CCR5 ligands can activate the canonical JAK/STAT pathway [214, 271, 272, 287, 349]. In HMBECs, HIV virions were also shown to activate STATs 1 and 3 and induce production of inflammatory cytokines. Several of these studies further demonstrated that HIV released from infected cells activated STATs [415, 420], potentially disrupting the blood–brain barrier. These studies also suggest that there may be differences in HIV-associated STAT activation between cell types, and that HIV may activate certain STATs in a JAK-independent manner. In contrast to the number of pathways activating STATs, the upregulation of MMPs, which are responsible for the degradation of extracellular matrix (ECM), appears to be largely mediated by p38 MAPK [277, 387, 422]. Gp120 mediated increases in MMPs have been shown in a number of cell types, including MDDC, T-cells, and astroglioma cells [277, 387, 423]. The signaling underlying this process is not clear, but it is likely regulated by the G-protein dependent and independent mechanisms described above. Together, these data further support the critical role of the MAPK cascade in mediating the effects of HIV on both target cells and surrounding cells.

### Changes in the actin cytoskeleton

Binding of gp120 to CCR5 and CXCR4 is also linked to alterations in the cytoskeleton, which may be important in both pre-and post-entry stages of infection. Both HIV and gp120 can activate Rho and Rac GTPases, predominantly Rho, cdc42, and Rac [281, 359, 424–426]. These GTPases are linked to cytoskeletal dynamics, including organization of actin and microtubules. Harmon et al., demonstrated the importance of these pathways in an astrocyte model of viral entry, showing that R5 gp120 initiates a signaling cascade, via G_αq_, that activates Rac1 and is required for viral fusion [281]. The need for activation of the G_αq_ cascade, particularly PKC, in early entry and replication events is supported by other studies that show inhibition of PKC delta significantly decreased HIV replication, but not entry, in hMDM [360]. Thus, co-receptor mediated changes in the cytoskeleton that allow viral fusion seem to require G_αq_ mediated activation of small GTPases.

Both X4 and R5 gp120 mediate viral fusion through changes in Rac and Rho GTPase activity, but X4 gp120 has specific effects on the actin cytoskeleton that are critical for infection of resting T cells [366]. Static cortical actin in resting T cells blocks productive infection, and gp120-CXCR4 signaling helps to overcome this inhibition through activation of cofilin, a cellular actin-depolymerizing factor critical for actin dynamics and viral nuclear migration [366]. The effects of gp120 on actin dynamics are dose and conformation dependent [366, 427]. At high doses, gp120 acts more like CXCL12, triggering rapid cofilin phosphorylation and actin polymerization, followed by cofilin dephosphorylation and actin depolymerization [366, 368]. At lower doses, gp120 is incapable of triggering rapid changes, instead mediating gradual cofilin dephosphorylation and actin depolymerization [366, 427].

Alteration of the actin cytoskeleton by other modulators have also been reported. WAVE2 is part of a multiprotein complex linking receptor signaling to actin nucleation and filament branching through Arp2/3. Gp120 triggers WAVE2 phosphorylation through both CXCR4 and CCR5, acting through early G_αi_ independent and late G_αi_ dependent mechanisms [428], and inhibition of Arp2/3 activity significantly attenuated HIV-1 nuclear migration and infection of CD4 + T cells [428]. Additionally, both R5 and X4 gp120 alter LIMK/cofilin signaling, which regulates actin depolymerization at the pointed (-) ends of actin filaments [365, 366, 428]. LIMK regulates cortical actin dynamics in CD4 + T-cells, and LIMK knockdown significantly inhibited early HIV viral DNA synthesis and CXCR4 internalization in these cells [365]. As both LIMK and WAVE2 are activated by Rac1 [359, 365, 429], and are key players in regulating actin dynamics [429], these data suggest small GTPase regulation of cofilin and ARP2/3 dynamics by gp120 allows HIV to hijack the actin cytoskeleton machinery to regulate actin treadmilling, promoting HIV nuclear migration.

Thus, co-receptor-mediated changes in key cytoskeletal proteins present a mechanism by which distinct signaling pathways mediate viral entry and replication in a number of different cell models. Co-receptor-mediated cytoskeletal alterations may also be important in establishing viral latency, particularly in T-cell populations [183, 367], although a discussion of this is beyond the scope of this review. While the precise signaling pathways and effectors mediating the effects of gp120 may vary, it seems clear that co-receptor signaling plays an indispensable role in both the replication of HIV and the effects of HIV on surrounding cells.

## Co-receptor signaling in HIV neuropathogenesis

There are two prevailing hypotheses regarding HIV neuropathology, the direct injury hypothesis and the bystander effect theory [105]. The vast majority of research examining these areas in the context of gp120-co-receptor signaling studies co-receptor activation via application or expression of specific gp120 proteins. These data show that gp120 mediated activation of both co-receptors contributes to both mechanisms of injury, as well as to the spread of CNS infection [49, 50, 386, 430–433]. While these theories are not mutually exclusive, we did not find any studies from the post-cART era that show gp120 levels reach concentrations high enough in the CNS or periphery to have any biological effects [172]. In addition, although multiple CNS cell types express both CXCR4 and CCR5, including neurons, astrocytes, and dendritic cells [18, 114, 240, 241], neurons do not express CD4 [402], and CD4 is generally thought to be required for efficient binding of gp120 to either CCR5 or CXCR4 [69, 70]. Thus, it remains unclear whether direct interactions between monomeric gp120 and neuronal co-receptors have physiological relevance, and it may be that HIV-mediated neuronal injury is largely due to indirect effects driven by low levels of active replication in microglia and perivascular macrophages [47, 48, 50, 172]. However, several in vitro studies have indicated a role for neuronal CCR5 or CXCR4 in the actions of gp120, suggesting there may also be a role for CD4 independent interactions in neuropathogenesis [47, 49, 162, 163, 216, 431, 434–436].

Given these data, we will only briefly discuss indirect effects of co-receptor signaling on neuronal injury and focus primarily on the role of the previously defined co-receptor signaling cascades and effectors in driving HIV neuroinflammation, as well as how external factors such as drug abuse can alter these events. As with our discussion on HIV-mediated co-receptor signaling, we will largely discuss studies that have relied solely on monomeric gp120 concentrations unlikely to be found in the CNS, as these represent the majority of the literature pertaining to this subject and provide valuable insight into how the specific activity and signaling pathways activated by discrete co-receptors can drive neuroinflammation and neuroHIV as a whole.

### Neuroinflammation

Activation of both CCR5 and CXCR4 by gp120 can induce the production of a number of inflammatory cytokines and chemokines [218, 273, 286, 387–390, 403–405, 437], increase the transmigration of inflammatory and infected monocytes and T-cells [414, 420, 425, 438], activate macrophage and glial populations [103, 284, 439], alter BBB integrity [277, 415, 438, 440] and induce oxidative stress, which can exacerbate these effects [423, 439, 441–443]. The majority of these events occur in infected and uninfected myeloid cells, including both transmigrated peripheral monocytes, hMDM, and CNS resident cells such perivascular macrophages and microglia [50, 101, 444–448]. Astrocytes may also be involved [112, 444, 449, 450]. All myeloid cells express both co-receptors, with much higher levels of CCR5 [18, 19], and in response to interactions with gp120 they produce cytokines and chemokines that increase inflammation and recruit additional immune cells to combat infection. The cytokines associated with activation of co-receptors include TNF-α, IL-1β, CCL2, CCL3, CCL4, CCL5, CXCL10, CXCL12, IL-6, and IL-8, many of which are induced by MAPK-dependent signaling events, particularly p38 MAPK [273, 282, 286, 390, 392, 393, 397, 403, 405, 414, 430, 446, 451]. Elevated concentrations of TNF-α are found in the brains and cerebral spinal fluid (CSF) of HAND patients [452–455] and upregulated IL-1β mRNA has been found in cognitively impaired individuals [456]. Both cytokines are early regulators of the inflammatory response, and their release leads to activation of immune cells such as macrophages and microglia [454]. IL-1β also activates astrocytes, stimulating NF-κB activity [457] and the production of other inflammatory cytokines, including TNFα [454]. These cytokines can also act on neurons, contributing to synaptic injury and suggesting a mechanism by which gp120-induced production of chemokines can promote both inflammation and neuronal dysfunction [154].

Co-receptor induced increases in TNF-α could promote increased monocyte migration and dysregulation of the BBB by inducing the expression of adhesion molecules such as ICAM on endothelial cells [458] and promoting the production of chemoattractants, such as CCL2 and CCL4 [50, 459], which are released from macrophages and microglia. While early studies suggested CCL4 could inhibit the interactions between CCR5 and gp120 [460, 461], this is unlikely to occur in vivo given the ability of HIV to utilize different conformations of CCR5 to escape inhibition by endogenous chemokines [293, 462]. Thus, increased production of CCL4 could further activate CCR5, increasing the production of chemokines and cytokines that both recruit infected monocytes into the CNS and draw uninfected CNS cells to the site of infection, thereby promoting the spread of HIV [97, 187, 463, 464]. A variety of additional factors, including other viral proteins, cell debris, and aberrant levels of neurotransmitters such as dopamine and glutamate [465–471], further enhance the release of some of these chemokines from macrophages and microglia. In the pre-cART era, increased levels of these chemokines were observed in the CNS of individuals suffering from HIVE [452, 472], though this is far less common in the era of cART.

The development of neuroHIV is also influenced by the interactions of gp120 with co-receptors on non-myeloid populations, including both CNS dendritic cells and endothelial cells [285, 403, 414, 420]. Many of these effects are induced by gp120-CCR5 interactions that release STATs and MMPs. For instance, gp120-CCR5 interactions on dendritic cells activate several signaling cascades including the p38 MAPK pathway, driving dendritic cell migration and the release of inflammatory cytokines via downstream STAT signaling [285, 403]. As the number of dendritic cells in the CNS is greatly increased during neuroinflammatory diseases [473], dendritic cell exposure to infected myeloid cells or viral proteins could drive the recruitment of more dendritic cells to vulnerable areas of the CNS, further contributing to neuroinflammation and neuronal dysfunction. Co-receptor-gp120 interaction on endothelial cells comprising the BBB can also result in increased myeloid cell recruitment, as several in vitro models of the BBB show that gp120 can drive increased monocyte migration and damage the BBB. This occurs via reduced expression of tight junction proteins and increasing expression of the inflammatory factors IL-6 and IL-8 [414, 415, 420, 425, 438, 474]. The precise mechanisms underlying these results are unclear, but is likely due to multiple mechanisms, including PI3K-mediated STAT activation [414, 415, 438], activation of Rac1 and other GTPases [425], and PKC activation [438].

The activation of MMPs, which are upregulated in the CNS of HIV-infected individuals [475–477], may also play a critical role in the recruitment of infected cells into the CNS. These factors, such as MMP9, are associated with inflammatory diseases and increased permeability of the BBB [423, 440]. Both T-lymphocytes and C6 astroglioma cells increased MMP9 secretion in a p38 MAPK dependent manner in response to R5 and X4 gp120 [277], and both HIV and gp120 also increase MMP9 secretion in macrophages [387]. Further, activation of MMPs is also associated with drug abuse, particularly the use of methamphetamine [387, 478]. This demonstrates one of many potential mechanisms by which substances of abuse can exacerbate HIV-associated neuropathology, with a number of additional potential overlaps between HIV and substance abuse discussed in subsequent sections. Overall, these and many other studies indicate that co-receptor-gp120 interactions act through a variety of distinct but overlapping signaling mechanisms, including p38 MAPK, PI3K, STAT and MMP activation, to participate in the inflammatory impact of HIV in the CNS. Although much progress has been made, further studies are needed to tease out the relative importance and role of these different pathways, and how they may work together to promote the production of inflammatory factors, migration of infected cells and other processes involved in neuroinflammation.

### Co-receptor mediated neuronal injury

Although the mechanisms by which HIV interactions with neuronal co-receptors can directly drive neurotoxicity are not clear, several studies indicate that glial cells and macrophages can mediate the neurotoxic effects of gp120 [50, 154, 386, 479–482]. For example, inactivation or depletion of macrophages and microglia abrogates gp120-mediated neurotoxicity [50, 480]; additionally, activation of CXCR4 on macrophages/microglia is a prerequisite for gp120 neuronal injury [50, 386, 481], suggesting these effects are driven by co-receptors on glial populations. Additionally, microglial activation is required for R5 gp120-induced synaptic degradation in primary cortical cultures [482], and X4 gp120-induced upregulation of ferritin heavy chain (FHC), which is associated with cognitive deficits, only occurred in neuronal/glial co-cultures [154]. Many of these neurotoxic effects are also seen in patients with HAND, suggesting that gp120-mediated effects play a role in the development of neurological disease [483, 484]. Thus, while shed gp120 may not play a direct role in HIV-driven neurotoxicity, co-receptor activation by HIV virions in CNS immune cells and glia is still likely to contribute to neuronal damage.

As mentioned previously, activation of p38 MAPK is central to these neurotoxic effects, and this also seems to be mediated through non-neuronal cells. Activation of p38 MAPK was found in gp120-treated mixed cortical cultures (containing neurons, astrocytes, and microglia) and this was dependent on the presence of microglia [386]. Increases in glial p38 MAPK were necessary for gp120-mediated neuronal apoptosis in mixed neuronal-glial cultures [386], and in rat cerebral cortical cultures, gp120 appeared to indirectly induce neuronal apoptosis via activation of the p38 MAPK pathway in macrophages and microglia [50]. Downstream of p38 MAPK, the activation of MMPs from infected macrophages has been demonstrated to contribute to gp120-induced neurotoxicity, as these proteins can cleave CXCL12 into a neurotoxic product aa5-67 CXCL12. This cleaved form of CXCL12 is unable to properly bind to CXCR4; instead, it stimulates an alternative receptor, CXCR3, promoting neurotoxicity [485]. Upregulation of MMPs and cleavage of CXCL12 have been reported in HIV + brains and is suggested to contribute to neuropathology in humans [486]. Additionally, changes in various inflammatory cytokines, such as TNF-α and IL-1β, may further indirectly promote neurotoxicity through a variety of mechanisms, including Ca^2+^ overload [484], alterations in dendritic spine length [153, 154], and axonal degeneration [479], all of which are associated with cognitive deficits, particularly in the cART era [104–106]. Thus, many of the same signaling cascades implicated in co-receptor mediated neuroinflammation may also indirectly contribute to neuronal damage via glial-neuron interactions. This suggests that advancing our understanding of co-receptor mediated neurotoxicity will require models that enable the careful dissection of cross cell type interactions.

### Substance abuse and co-receptor-mediated neuropathology

Substance use disorders (SUD) have been comorbid with HIV infection since the start of the epidemic. Rates of SUD vary widely by region, substance, and specific sub-population, but are found in roughly in 9–48% of HIV-infected individuals globally, compared to 0.7–8.6% of the population as a whole [487–495]. There are particularly high rates of HIV infection among individuals who inject drugs—twenty-two times higher than in the general population [487, 488, 496]. The impact of SUD is especially important when considering neuroHIV, as the use of addictive substances is associated with increased neuropsychiatric comorbidities and is strongly correlated with cognitive decline, even with effective cART [497–500]. This is likely because use of illicit substances can promote neuroinflammation and alter the progression of HIV-associated neuropathology [501–504], increasing HIV replication and dysregulating cytokine secretion and other immune functions in myeloid cells such as macrophages and microglia [502, 504–507]. These effects can add to or synergize with the impact of HIV virions and viral proteins, including gp120, and host factors, exacerbating inflammation and damage to the BBB and recruiting more myeloid cells to enhance the spread of infection. Psychostimulants and opioids represent the major drug classes associated with SUDs in PLWH and have subsequently been the most studied in relation to gp120 and HIV neuropathogenesis.

Stimulants include methamphetamine (Meth), cocaine, and prescription drugs used to treat conditions such as ADHD, all of which act by directly increasing CNS dopamine [508–510]. Dopamine, and by extension substance abuse, has been shown to modulate HIV infection, replication and inflammation, as well as alter the efficacy of the CCR5 entry inhibitor Maraviroc [66, 373, 465, 509, 511–513], although a comprehensive discussion of the role of dopamine in HIV neuropathogenesis is beyond the scope of this review. However, studies also show that stimulants can directly synergize with gp120 to exacerbate neuropathology [387, 408, 437, 514–520]. Injection of cocaine and gp120 into rodent brains induced higher levels of apoptosis than either treatment alone [518], and treatment of rat primary neurons with gp120 and cocaine also enhanced apoptosis through an apoptotic pathway involving intracellular ROS production, mitochondrial membrane potential loss, and activation of the NF-κB and ERK, p38 and JNK/SAPK signaling pathways [514]. While this study did not demonstrate a direct role of co-receptors, these signaling pathways are activated by HIV interactions with co-receptors [273, 277, 282–286, 363, 385–390, 406], suggesting HIV-mediated activation of co-receptors on surrounding glia could indirectly exacerbate the effects of these drugs. This is further supported by studies demonstrating that cocaine and gp120 together may enhance microglial neurotoxicity, as data show cocaine + gp120 alters energy metabolism and AMPK expression to impair the function of CHME-5 microglia [521]. In transgenic rats expressing gp120, long term exposure to both gp120 and Meth synergized to impair learning and memory, dysregulate the components of GABAergic and glutamatergic neurotransmission systems and induce a loss of neuronal dendrites and presynaptic terminals in the hippocampus [522]. Learning deficits induced by Meth and gp120 exposure were also seen in gp120 + transgenic mice, with similar patterns of impairment as seen in HIV infected Meth users [523]. Moreover, exposure to both Meth and gp120 induced greater PBMC transmigration and greater decreases in trans-endothelial resistance and expression of tight junction proteins than either meth or gp120 alone [515].

These affects may be further amplified through the direct interaction of stimulants with CCR5. Cocaine can increase mesolimbic CCR5 expression, and the conditioned place preference behaviors associated with cocaine are reduced by the use of maraviroc, a CCR5 antagonist [524]. Meth has been shown to increase CCR5 expression in cultured macrophages and in the myeloid cells of SIV infected macaques [507, 525–527]. We and others have shown that dopamine can increase the expression of certain CCR5 conformations on the surface of human macrophages [498] and THP-1 cells [528]. As small changes in surface expression of CCR5 can induce substantial changes in infection [529–532], drug-associated changes in CCR5 expression or conformation could promote greater levels of viral infection, leading to increased levels of neurotoxic viral particles and an expanded viral reservoir. Together, these data suggest that the use of stimulants and the dopamine release they induce may synergize to increase infection and inflammation in CNS cells, increasing the spread of HIV and expanding the CNS reservoir while also exacerbating the development of neuroinflammation.

Opioids are also commonly abused in HIV-infected populations and can exacerbate the symptoms of HAND [316, 533–535], potentially through interactions with both CXCR4 and CCR5 on glia and neurons. Opioids can increase both CCR5 expression and the expression of toll-like receptors in astrocytic model systems, potentially increasing the magnitude of the inflammatory response to gp120 [437, 536–538]. Opioid receptors may also heterodimerize with CCR5, with some studies showing mu opioid receptor (MOR) agonists reduce the chemotactic effects of CCR5 [539, 540], while others show that CCR5 ligands dampen the analgesic effects of MOR agonists [541, 542]. Stimulation of the MOR can inhibit homeostatic CXCR4 signaling in the CNS via the FHC protein [543], and stimulation of neurons co-expressing CXCR4 and MORs with morphine or the MOR agonist DAMGO inhibited intracellular pathways activated by CXCL12, preventing CXCL12-mediated neuroprotection and CXCR4 phosphorylation [543, 544]. This results in long-lasting inhibition of the receptor that is distinct from more common opioid-chemokine cross regulatory mechanisms including heterologous desensitization [542] and receptor dimerization [545]. Further, HIV gp120 can upregulate FHC in neuronal/glial cocultures via an IL-1β dependent mechanism [154], suggesting opioid use and HIV infection may act through overlapping or shared mechanisms to induce cognitive impairment. Several studies have also indicated that CCR5 plays a central role in modulating tat-morphine interactions, with the loss of CCR5 blocking tat-induced neurotoxicity, morphine tolerance and the release of pro-inflammatory cytokines, including CCL2 [448, 541]. This suggests crosstalk between CCR5 signaling and MOR signaling could lead to unique signaling effectors that exacerbate neuroinflammation and neuronal damage. Overall, these data indicate that the interactions between drugs of abuse and co-receptors can lead to a variety of detrimental effects, increasing both HIV infection within the CNS and contributing to neuropathology. Defining these processes more precisely is critical, as the mechanism(s) by which distinct substances of abuse exacerbate neuroHIV are unclear, hindering development of therapeutics specific to the vulnerable and growing population of HIV-infected drug abusers.

## Co-receptors as targets in antiretroviral therapy

Co-receptors are essential for HIV entry and therefore make an attractive target for anti-retroviral therapeutics. Blocking cell entry prevents viral replication, and when combined with other drugs this can achieve sustained suppression, halting disease progression. Further, the role of co-receptor signaling in modulating neuroinflammation suggests that targeting the downstream signaling events may reduce the inflammatory effects of HIV, particularly in combination with other antiretrovirals. However, despite more than 30 years of antiretroviral development and numerous claims that co-receptor targeting was an emerging, promising therapy [546–549], there is currently only one antiretroviral that acts on a co-receptor, the CCR5 inhibitor Maraviroc. There are several CCR5 inhibitors that were halted or are in ongoing, late-stage clinical trials [44], and there is a CXCR4 inhibitor, Plerixafor (AMD3100), but it is only approved for mobilization of hematopoietic stem cells, due to severe off-target effects associated with CXCR4 inhibition [42, 550]. A number of small molecular inhibitors and monoclonal antibodies have also been investigated as potential methods for blocking HIV interactions with CCR5, but most have not progressed past phase II trials, and none to date have been approved by the FDA [37, 547, 551, 552], although the monoclonal antibody leronlimab (PRO 140) has been granted fast-track status [551]. And although they do not target the co-receptors directly, the anti-CD4 antibody Trogarzo, the gp120-targeting attachment inhibitor Fostemsavir, and the fusion inhibitor Enfurvitide also inhibit the entry process mediated by CCR5 and CXCR4 [553–555].

There are a number of reasons co-receptors are difficult to target, including the ability of HIV to utilize multiple different conformations [293, 300] and interference of therapeutics with homeostatic signaling leading to off-target effects [43, 556]. The latter is particularly problematic for targeting CXCR4, as the essential function of CXCR4 in numerous homeostatic processes precludes the development of inhibitors targeting this receptor [43, 557]. Targeting CCR5 and CXCR4 are both associated with their own unique set of challenges; therefore, we will address these challenges and the current state of therapeutics separately. We will then address how we can utilize overlaps in the signaling processes associated with HIV binding to both receptors to potentially develop therapies to ameliorate both HIV infection and associated neuroinflammation.

### CCR5

Early in the HIV epidemic, it was observed that individuals homozygous for the CCR5-$\Delta$ 32 mutation, which renders CCR5 nonfunctional, were resistant to HIV infection and lacked significant immunological defects [558, 559]. The importance of CCR5 as an antiretroviral target was confirmed when the “Berlin patient” received a stem cell transplant from an individual homozygous for CCR5-$\Delta$ 32 and was functionally cured of HIV [560]. Another patient receiving the same treatment is also in successful long-term remission from HIV infection [561], and CCR5-targeted drug Maraviroc is an effective antiretroviral. However, Maraviroc is highly susceptible to resistance mutations [45], necessitating the development of alternative methods of targeting CCR5 [562]. Unfortunately, effectively interfering with viral binding to CCR5 is much more complex than had initially been anticipated. Early structural studies showed gp120 binds to the N-terminus of CCR5, the same region targeted by endogenous ligands, and in vitro studies showing that CCL3, CCL4, and CCL5 have potent antiretroviral activity against R5 viruses [190, 460–462, 563]. However, newer studies suggest gp120 interacts with CCR5 differently than do natural ligands [257, 292–294, 300] and the antiretroviral activity of endogenous CCR5 ligands has not replicated across cell systems or in vivo [260, 299, 564].

This may be due to the capacity for gp120 to efficiently interact with CCR5 in multiple structural conformations, whereas natural ligands and inhibitors such as Maraviroc can only target specific conformations [293, 294, 298, 300, 301]. Mutational studies and those using antibodies targeting distinct CCR5 conformations confirm that CCR5 exists in multiple conformations and oligomerization states on a single cell, and these different states alter the binding affinity of multiple ligands [215, 257, 268, 292, 294, 299, 300, 565]. Indeed, changes in CCR5 conformation or mutations in gp120 can lead to a loss of efficacy for Maraviroc due to altered binding interactions between CCR5 and gp120 [45, 293, 300, 565]. In addition, antiretrovirals that bind directly to CCR5 can still induce downstream signaling events, many of which can potentiate the impact of HIV on the CNS [37, 260]. For these reasons, the use of cognate CCR5 ligands is not considered a viable method to block HIV binding to this receptor.

This has led to an interest in developing biased ligands or small molecules that promote the internalization of CCR5 or bias signaling pathways to prevent HIV entry without stimulating downstream signaling [38, 39, 215, 256, 262, 566]. Several different groups have designed effective chemokine analogues that target CCR5, promoting the internalization and delaying the recycling of CCR5, rather than competing with other ligands for the binding site [38, 214, 256, 258, 260, 290, 302]. These molecules have up to 200 times the inhibitory potency of Maraviroc on the viral entry process. Perhaps more importantly, they may not promote intracellular signaling cascades, instead biasing the receptor towards association with β-arrestins and internalization [38, 39, 256, 262, 263, 566]. These analogues may be more effective than binding inhibitors like Maraviroc because they reduce the surface levels of CCR5, diminishing the impact of changes in gp120 binding regions or CCR5 conformations [256]. Additionally, it is possible that these molecules could exploit the ability of CCR5 to oligomerize with other receptors; a number of in vitro studies have suggested that homo- or hetero-oligomerization of CCR5 can block HIV infection and replication [244, 269, 270]. However, the impact of the formation of these oligomers on chemokine-induced signaling and their in vivo relevance is incompletely understood and requires a more complete understanding of chemokine and HIV-induced signaling.

### CXCR4

While blocking CCR5 has been considered a more promising strategy for inhibition of viral entry and replication, an increasing number of patients are developing X4 tropic or dual-tropic viral strains that can bind to CXCR4 [557]. This suggests that targeting CXCR4 may be particularly beneficial for patients in the later stages of infection. Early chemical screening and medicinal chemistry efforts identified several CXCR4 antagonist peptides, including Plerixafor (AMD3100), one of the first CXCR4 antagonists to enter clinical trials for anti-HIV activity [550]. Although AMD3100 appears to have a distinct binding site from the region occupied by the N-terminus of CXCL12, it still produced severe off-target effects, including cardiotoxicity, and these trials were terminated [567]. Several derivatives of AMD3100 have been developed that inhibit CXCR4 at sub-nanomolar concentrations; however, clinical utility of these compounds is limited due to their lack of oral bioavailability, which is related to their high positive charge at physiological pH [556]. Current studies have moved away from the early peptides and are using natural CXCR4 ligands as design templates, similar to the chemokine analogue-based strategies described for CCR5. The N-terminus of CXCL12 has been shown to be essential for CXCR4 recognition, signal transduction, and antiretroviral activity; however, peptides targeting this region are less potent than native CXCL12, limiting their clinical effectiveness [556]. In a similar strategy, the N-terminus of another chemokine, viral macrophage inflammatory protein-II (vMIP-II) from human herpesvirus-8, was used as the design template for various classes of highly potent and selective CXCR4 peptide antagonists. While these peptides display high CXCR4 affinity, anti-HIV activity, and the ability to mobilize hematopoietic stem cells in mice, they are still in the preclinical phase [568].

There are two primary challenges associated with developing CXCR4-targeting therapies, accommodating receptor oligomerization and minimizing undesirable side effects due to the normal, homeostatic functions of CXCR4. The ability of receptors to affect one another through oligomerization makes it essential to consider the in vivo state of CXCR4, particularly because CXCR4 may homodimerize in both the absence [569] or presence of ligand [244, 570] soon after protein translation. Additionally, CXCR4 may also form heterodimers with other chemokine receptors like CCR2 [244, 570], as well as non-chemokine receptors, including CD4, opioid receptors, and glycoproteins [351]. Although some studies have suggested that the induction of these dimers could be used as a strategy to target both R5 and X4-mediated infection [244, 269], the functional consequences of oligomerization are not fully understood. This makes it difficult to predict the effects of pharmacological compounds if they do not target oligomerization states CXCR4 exist as in vivo. However, oligomerization may also be beneficial, designing drugs that target unique receptor complexes, though this will be technically more challenging.

The biggest challenge in the development of CXCR4-targeted HIV entry inhibitors is overcoming undesirable side effects that result from inhibition of CXCR4, as this receptor has essential roles in numerous homeostatic functions in the periphery and CNS. Studies on two of the CXCR4 antagonists brought into clinical testing (AMD3100 and AMD11070) were terminated early due to toxicity [557]. Thus, it is essential to develop molecules with potent anti-HIV activity, while preserving CXCL12 signaling. A number of small molecules and nanobodies have shown promise in vitro in blocking HIV binding while preserving CXCL12 interactions, but it remains unclear if this will translate to clinically useful drugs with an adequate safety profile in vivo [43, 571, 572]. A more promising way to address these off-target effects is the development of allosteric agonists that can activate CXCR4 in the presence of other CXCR4 antagonists and antibodies [43]. These allosteric agonists, such as RSVM and ASLW [331], may be useful in combination with small-molecule antagonists to block viral entry but still maintain homeostatic activation of CXCR4. However, this will require further understanding of the intracellular signaling mediating both homeostatic receptor activation and viral entry.

### Strategies to target co-receptor signaling pathways

While strategies to target CCR5 and CXCR4 via small molecules, antibodies, and chemokine analogues are promising, none of these holds promise in blocking both receptors concurrently. Dual co-receptor antagonists have been described [549], but the majority have only undergone in vitro and in silico evaluation, and many show inadequate safety profiles and poor pharmacokinetics. Given differences in the requirements for CCR5 and CXCR4 under homeostatic conditions, effective inhibition may require different approaches to ensure the functionality of CXCR4 in particular. An alternative method to target both co-receptors may lie in drugs targeting common downstream signaling effectors induced by HIV-co-receptor interactions [41]. Although such drugs may not serve as antiretrovirals on their own, as they do not sufficiently block the viral life cycle, they could potentially act to ameliorate co-receptor mediated neuroinflammation and pathology. Given the prominent role of persistent neuroinflammation in driving neuropathology in the cART era, there is a critical need to target mechanisms of inflammation within the CNS.

CCR5 and CXCR4 have a number of common pathways, with several specific effectors such Pyk2 and p38 MAPK that are involved in a number of HIV-associated pathologies. These effectors are also involved in other diseases, including cancer [573, 574], so a number of inhibitors are already on the market, or in drug pipelines, and could be repurposed as HIV drugs. Inhibitors of tyrosine and MAP kinases may have anti-HIV activity [575, 576], and p38 MAPK inhibitors have been examined as potential therapeutics in several inflammatory diseases and cancers [577]. A recent primate study using a p38 MAPK inhibitor in combination with cART showed this combination reduced markers of immune activation, although only in the periphery [576]. But despite over 20 candidates in clinical trials, no specific MAPK inhibitors have been clinically approved [577], likely due to the substantial number of signaling pathways and pathological and physiological processes in which this kinase plays a role. A more promising avenue may be to focus on tyrosine kinase inhibitors (TKIs), which are directed against Src family kinases [573, 578]. The Src family of kinases is linked to both the PI3K/AKT and MAPK signaling cascades via multiple mechanisms [410, 412, 413], and, in hMDM, can mediate changes in IL-1β and TNFα in response to R5 gp120 [264, 286]. These have been examined largely in the context of inhibiting replication and the establishment of the viral reservoir in T-cells [573, 578], and TKIs such as dasatinib can both protect against HIV infection in humanized mice [579] and inhibit HIV replication in T-cells [580]. This suggests TKIs could be a useful adjuvant during early HIV infection to prevent establishment of viral reservoirs [581], and the centrality of both PI3K and MAPK signaling to co-receptor mediated neuroinflammation suggests they could be promising targets in the CNS as well. Despite the fact that some of these therapeutics, such as dasatinib, have been approved for some cancers [582], they have not been sufficiently proven as an effective anti-HIV therapeutic [573]. Additionally, these studies have not addressed the potential use of these therapeutics in specifically ameliorating CNS pathology driven by HIV-co-receptor interactions, despite in vitro evidence demonstrating a role for Src and its regulation of the MAPK and PI3K cascades in these effects [264, 286, 412, 413].

## Conclusion

A huge body of work indicates co-receptor signaling pathways activated by HIV are not only critical to the viral entry and replication process, but also to the broader neuroinflammatory and neurotoxic impact of HIV in the CNS. There have been numerous attempts to develop therapeutics targeting CXCR4 and CCR5, but most of these candidates did not or have not yet advanced out of trials, and many failed due either lack of efficacy or serious side effects [43, 583–587]. The lack of success highlights the gap in our knowledge regarding how these effectors are activated by gp120 and natural ligands, their role in mediating physiological CCR5 and CXCR4 signaling, and the impact of their downstream signaling effectors on homeostatic and pathological processes. The signaling cascades initiated through both CCR5 and CXCR4 have a wide array of downstream consequences, mediated by a variety of overlapping pathways triggered by both HIV and chemokine interactions. Defining which of these pathways is associated with a specific ligand, G-protein, set of downstream effectors, and/or receptor conformations is critical to understanding of how to target them. The emerging data suggesting that discrete receptor conformations induce distinct downstream events, and that these events are also cell-type specific, speaks to a need for comprehensive examinations of the conformations targeted by specific ligands, the signaling pathways and kinetics of each signaling event, and the downstream consequences for the specific cell and body as a whole. Filling in this knowledge gap is essential to the development of therapeutics targeting specific signaling pathways and highlights the need for rigorous studies in a more physiologically relevant context.

It is important to note that the majority of studies discussed here have been performed using highly artificial conditions. Thus, while they suggest a critical role for co-receptors, they preclude drawing a definitive conclusion regarding the impact of co-receptor signaling on HIV neuropathogenesis. This further highlights the need for future studies to make use of physiologically relevant cell types, and take advantage of new technologies, such as 3D culture systems and iPSC-derived primary cells, as well as animal models that more closely parallel the human immune system. Many of the neuroinflammatory effects of HIV-co-receptor interaction involve multiple cell types and show distinct activity in co-cultures relative to monocultures. Therefore, the development and use of mixed culture systems and organoids using combinations of neurons, glia and other myeloid cells will be critical to better mimic the interactions in the CNS. Combining data from these systems with more reductionist, single cell models that capitalize on newer technologies such as CRISPR and single cell genomic analyses such as single cell RNA sequencing (scRNAseq) and single cell Assay for Transposase Accessible Chromatin (scATACseq), will enable targeted analyses of changes in the expression, transcription and or function of specific effectors within different signaling pathways. Moreover, pairing these assays with conformational studies will enable precise assessment of the effects of specific conformational changes on HIV binding and signaling though CXCR4 and CCR5. These new systems should be combined with experimental consistency and physiological accuracy in regard to the use of HIV concentration and treatment paradigms. This is particularly true in the use of monomeric gp120 versus intact HIV virions, given the lack of a demonstrable role of gp120 in driving neuroHIV in the cART era. The use of monomeric gp120 has been valuable in a number of areas, including studies highlighting the complexity of signaling pathways associated with HIV activation of co-receptors. However, the field needs to better address signaling in response to HIV virions, as this may reveal distinct differences in co-receptor engagement and point towards more physiologically relevant signaling effectors that can be targeted.

A better understanding of the dynamic interaction between HIV and the CXCR4 / CCR5 co-receptors should enable the efficient development of ligands that precisely target only the signaling processes involved in entry. More broadly, both CXCR4 and CCR5 affect a wide array of diseases and homeostatic processes, from cancer to cardiac disease, indicating that effectively targeting co-receptors and manipulating their signaling could be a useful tool in the treatment of a number of diseases. Thus, these same studies, experimental pipelines and drug candidates will be used to develop therapeutics that not only inhibit the effects of co-receptors on HIV neuropathogenesis but could potentially be used to treat a number of other important human diseases.

## Data Availability

Not applicable.

## Abbreviations

HIV: Human immunodeficiency
AIDS: Acquired immunodeficiency syndrome
PLWH: People living with HIV
ART: Antiretroviral therapy
GPCR: G-protein coupled receptor
CNS: Central nervous system
cART: Combined antiretroviral therapy
HAND: HIV-associated neurocognitive disorders
BBB: Blood–brain barrier
PFC: Prefrontal cortex
HIVE: HIV encephalitis
MAPK: Mitogen activated protein kinases
PI3K: PI3 kinase
GRK: GPCR kinases
cAMP: Cyclic AMP
PLCβ: Phospholipase C-beta
PIP_2_: Phosphatidylinositol 4,5-bisphosphate
DAG: Diacylglycerol
IP_3_: Inositol triphosphate
Ca^2^^+^: Calcium
TGN: Trans-golgi network
PTX: Pertussis toxin
JAK: Janus kinases
STAT: Signal transducers and activators of transcription
AMPK: AMP-activated protein kinases
MMPs: Matrix metalloproteinase
NT: N-terminal
ECL: Extracellular loop
hMDM: Human monocyte derived macrophages
MDDC: Monocyte derived dendritic cells
HBMEC: Human brain microvascular endothelial cells
TCR: T-cell receptor
ECM: Extracellular matrix
CSF: Cerebral spinal fluid
BDNF: Brain derived neurotrophic factor
KCC2: K^+^-Cl-cotransporter 2
Rb: Retinoblastoma gene
FHC: Ferritin heavy chain
FKN: Fractalkine
Meth: Methamphetamine
ROS: Reactive oxygen species
MOR: Mu opioid receptor
